# On the Role of Spin–Orbit Coupling and State-Crossing Topography in the Nonradiative Decay of Ir(III) Complexes

**DOI:** 10.1021/acs.jpclett.5c01776

**Published:** 2025-08-25

**Authors:** Iván Soriano-Díaz, Ilya D. Dergachev, Sergey A. Varganov, Enrique Ortí, Angelo Giussani

**Affiliations:** † Institute for Molecular Science (ICMol), 16781 Universitat de València, Catedrático José Beltrán 2, 46980 Paterna, Spain; ‡ Department of Chemistry, 6851 University of Nevada, Reno, 1664 North Virginia Street, Reno, Nevada 89557-0216, United States

## Abstract

A pillar of our current understanding of the photoluminescence of Ir­(III) complexes is the assumption that the population of triplet metal-centered states determines an efficient nonradiative decay to the ground state minimum. Based on that assumption, the energy separation between the emitting state and the minimum-energy crossing point of the triplet metal-centered and ground states has been employed as a key variable for evaluating the ability of Ir­(III) complexes to decay nonradiatively. We demonstrate that the strong spin–orbit coupling between the triplet metal-centered and ground state of Ir­(III) complexes, together with the sloped topography of their crossing, leads to a significant energy separation between the two states, resulting in a reduced rate of nonradiative ground state recovery. Therefore, we propose that the role of metal-centered states is defined by the tendency of the excited state population to remain trapped in the metal-centered minima.

## Graphical abstract

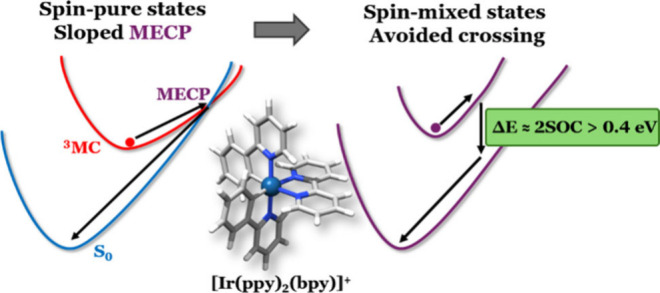

Ir­(III) transition-metal complexes (Ir3-TMCs),^1–8^ which are characterized by a d^6^ electronic configuration and strong spin–orbit coupling (SOC), are fundamental for electroluminescent devices and photodynamic therapy (see Sections S1 and S2 of the Supporting Information). Because for both applications slow nonradiative decay is required, much effort has been devoted to determining the main nonradiative processes at play, with the ultimate goal of devising chemical modifications that minimize them. In general, two intramolecular nonradiative decays are considered.^9–11^ The first is the intersystem crossing (ISC) from the emitting triplet minimum to the ground state (S_0_). The second is the population of triplet metal-centered states (^3^MC), which are expected to efficiently mediate a nonradiative decay to the ground state.

^3^MC states of Ir3-TMCs are characterized by equilibrium structures in which at least one of the coordinating metal bonds is dissociated (Section S2). This dissociation causes a massive increase of the ground state energy at the ^3^MC minima and the presence of energetically and geometrically nearby ^3^MC/S_0_ minimum-energy crossing points (MECPs).^12–16^ It is the existence of such accessible MECPs, together with the large SOC, that leads to the assumption that ^3^MC states efficiently drive the population back to S_0_. Most of the theoretical understanding of the photophysics of Ir3-TMCs is based on the validity of this assumption, relating an increase/decrease in the emission quantum yield with a decrease/increase in the accessibility of ^3^MC states.

The presumed highly efficient decay of ^3^MC states has led the scientific community to employ transition state theory (TST) to characterize ^3^MC-mediated nonradiative decay, treating the ^3^MC/S_0_ MECP as an analogue of the transition state (TS) geometry.^9–11,14,17^ This implies that the probability of decay to S_0_ is equal to unity at MECP, as in TST the probability of reaction is unity once the system reaches a TS. Here we demonstrate that for Ir3-TMCs the idea that the ^3^MC/S_0_ MECP mediates an efficient repopulation of the Franck–Condon region cannot be assumed to be in general valid.

As a representative example, we focus on [Ir­(ppy)_2_(bpy)]^+^ (where Hppy is 2-phenylpyridine and bpy is 2,2′-bipyridine), which is an archetype of the [Ir­(C^N)_2_(N^N)]^+^ cyclometalated Ir­(III) complexes used in electroluminescent applications.^2,3^ Our results are general and apply to any Ir3-TMCs. The emitting T_1_ state of [Ir­(ppy)_2_(bpy)]^+^ has metal–ligand charge transfer (MLCT) character and displays an equilibrium structure similar to the S_0_ minimum.^13,18^ From T_1_, the processes determining the efficiency of light emission are supposed to start: phosphorescent emission, direct ISC to S_0_, and population of the ^3^MC states with the subsequent transfer back to S_0_ via the ^3^MC/S_0_ MECPs. The latter process is a T_1_-to-S_0_ decay mediated by the ^3^MC/S_0_ MECP, because, even if the nature of the triplet state changes from MLCT to MC, the involved potential energy surface (PES) is always the adiabatic T_1_. Two types of ^3^MC states have been identified in [Ir­(ppy)_2_(bpy)]^+^.^13^ The axial ^3^MC_ax_ state, which is characterized by a minimum structure (^3^MC_ax_)_min_ displaying the dissociation of one Ir–N_ppy_ bond, and the equatorial ^3^MC_eq_ state, where an Ir–N_bpy_ bond is broken in its equilibrium geometry (^3^MC_eq_)_min_. Both bonds are present in the octahedral structures of the emitting T_1_ MLCT and S_0_ minima, so the evolution from the former to the latter, through the ^3^MC/S_0_ MECP, implies the stretching of an Ir–N bond up to dissociation and then the reformation of the very same bond (Figure 1). Therefore, an Ir–N distance plays the role of the reaction coordinate connecting the T_1_ minimum and the ^3^MC/S_0_ MECP. This means that both the initial and final minima (the emitting T_1_ and the S_0_ minima) are located along the same direction relative to the MECP. Borrowing from the terminology associated with conical intersections (CIs), we can say that the ^3^MC/S_0_ intersection is sloped, with the gradients of two states pointing in the same direction.^19–23^ The energies, structures, and orbital analysis of the described paths involving the ^3^MC_ax_ and ^3^MC_eq_ states are present in Section S3. These data have been previously obtained at the B3LYP/def2-SVP CPCM (CH_2_Cl_2_) level,^13^ and now also using the PBE0 functional.

**Figure 1:**
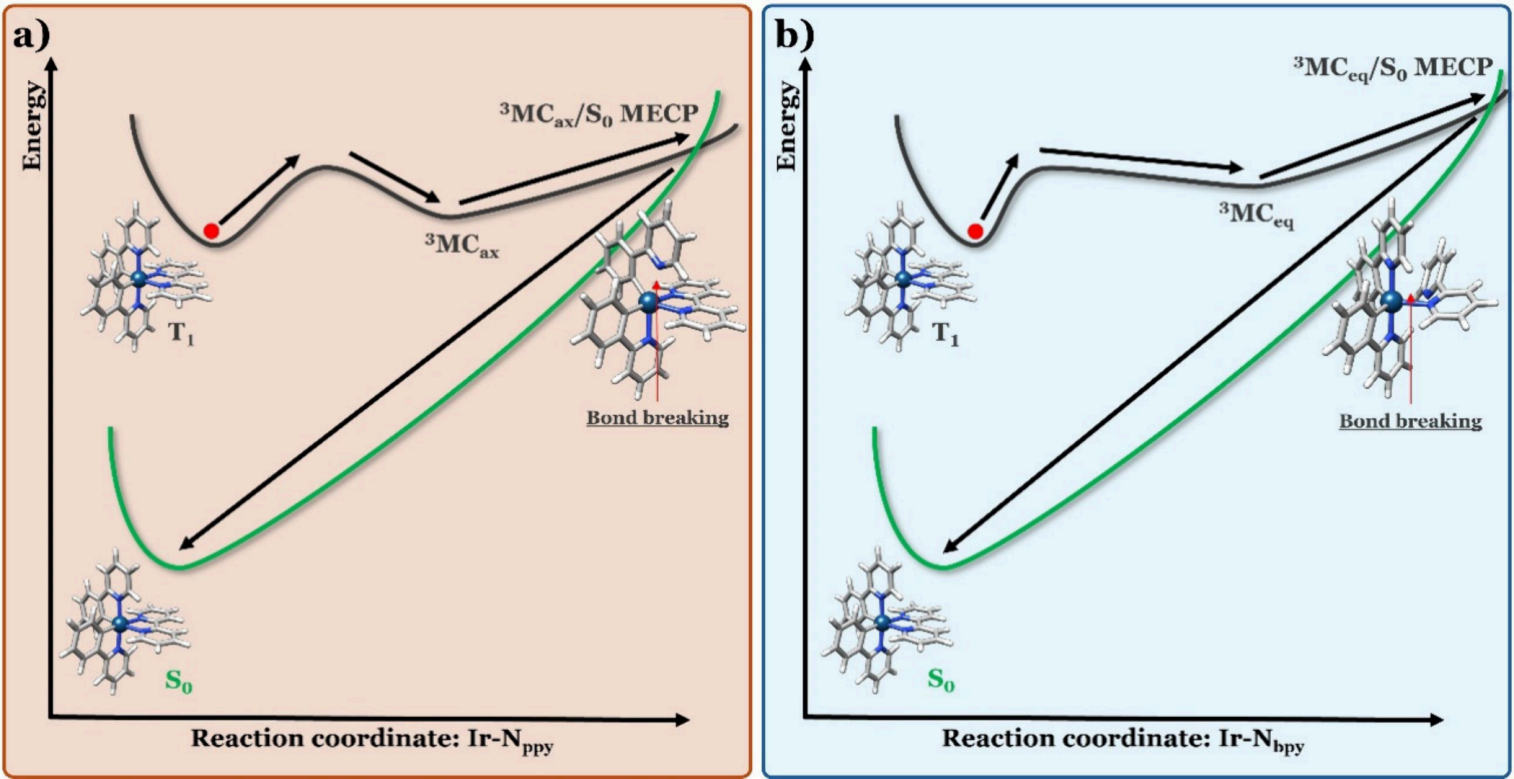
Nonradiative decay of [Ir­(ppy)_2_(bpy)]^+^ from the T_1_ minimum to the S_0_ minimum mediated by (a) the ^3^MC_ax_/S_0_ MECP and (b) the ^3^MC_eq_/S_0_ MECP.

The topography of the MECP and the value of the SOC between the ^3^MC and S_0_ states are key variables that strongly determine the probability of nonradiative decay. To illustrate this most clearly, we first need to look at the theory behind nonadiabatic events (NAEs) (Section S4).^21,24–26^ Let us first analyze the case of an NAE between two adiabatic electronic states of pure spin having the same spin multiplicity (i.e., an internal conversion (IC)). Expanding the wave function in the basis of these two spin-pure adiabatic electronic states, it is possible to show that what causes the passage of population from one PES to the other are the nonadiabatic couplings (NACs) (eq S8 and eq 1). Since, according to the generalized Hellman–Feynman theorem, the value of the derivative coupling between two states, which is the main component of the corresponding NAC, is inversely proportional to their energy separation (eq S10 and eq 3), the closer the states are, the more probable is the NAE, and that is why CIs are key in photochemistry.^19,22^

In the case of considering just the S_0_ and first singlet excited state, S_1_, the discussed equations will read as follows.

1 $$i{\mathrm{\psi ̇}}^{S_{1}}\left(R,t\right)=\left({\mathrm{T̂}}_{R}+V^{S_{1}}\left(R\right)\right)\psi^{S_{1}}\left(R,t\right)+{\mathrm{\Lambda ̂}}^{S_{0}S_{1}}\psi^{S_{0}}\left(R,t\right)$$

2 $${\mathrm{\Lambda ̂}}^{S_{0}S_{1}}≅-\left\langle S_{0}\left|{\mathrm{\nabla ̂}}_{R}\right|S_{1}\right\rangle \frac{1}{M}\nabla_{R}$$

3 $$\left\langle S_{0}\left|{\mathrm{\nabla ̂}}_{R}\right|S_{1}\right\rangle =\frac{\left\langle S_{0}\left|{\mathrm{\nabla ̂}}_{R}{Ĥ}_{el}\right|S_{1}\right\rangle }{V^{S_{1}}\left(R\right)-V^{S_{0}}\left(R\right)}$$

where ψ̇^S_1_^(**R**, *t*) is the time derivative of the nuclear wave function associated with state S_1_, *ψ* ^S_0_^(**R**, *t*) is the nuclear wave function associated with S_0_, *Λ̂* ^S_0_S_1_^ is the NAC between S_0_ and S_1_, *V* ^S_1_^(**R**) and *V* ^S_0_^(**R**) are the S_1_ and S_0_ PESs, respectively, ⟨S_0_|∇̂_*R*_|S_1_⟩ is the derivative coupling between S_0_ and S_1_, and *T̂*_R_ is the nuclear kinetic energy operator.

Let us now expand our wave function in the basis of two spin-pure adiabatic electronic states having different spin multiplicities, for example, a triplet and a singlet state. Such states are also called spin-diabatic states.^27–31^ Repeating the previous mathematical manipulations, it now appears that the corresponding NAC is zero due to the orthogonality of the different spin wave functions, and that what couples the two states and drives an NAE is the SOC term (see eq 4), assuming it is included in the Hamiltonian.^32–34^ Considering only the S_0_ and T_1_ states, the coupling equation can be written as follows.

4 $$i{\mathrm{\psi ̇}}^{T_{1}}\left(R,t\right)=\left({\mathrm{T̂}}_{R}+V^{T_{1}}\left(R\right)\right)\psi^{T_{1}}\left(R,t\right)+H_{SOC}^{S_{0}T_{1}}\psi^{S_{0}}\left(R,t\right)$$

where *ψ̇* ^T_1_^(**R**, *t*) is the time derivative of the nuclear wave function associated with state T_1_ and ${Ĥ}_{SOC}^{S_{0}T_{1}}$ is the SOC between S_0_ and T_1_.

Let us again use the two spin-pure adiabatic electronic states of different spin symmetry (a singlet and a triplet) as a basis for the expansion of the wave function, but instead of using them as they are, let us use the combination resulting from the diagonalization of the electronic Hamiltonian including the SOC term (eq 5).

5 $$\left(\begin{array}{ll} V^{S_{0}} & H_{SOC}^{S_{0}T_{1}}\\ H_{SOC}^{S_{0}T_{1}} & V^{T_{1}} \end{array}\right)\overset{\mathrm{diagonalization}}{\Rightarrow }\left(\begin{array}{ll} V^{SM_{0}} & 0\\ 0 & V^{SM_{1}} \end{array}\right)$$

We call the resulting states, no longer of pure spin multiplicity, spin-mixed, or spin-adiabatic states. The energy separation between these spin-adiabatic states depends on the magnitude of the SOC between the original spin-diabatic states. If we expand the wave function in the basis of such spin-adiabatic states, they will be again coupled by NAC, which determines an NAE, now computed over the two spin-mixed states (see eq 6). Again, the value of the NAC is inversely proportional to the interstate energy separation; therefore, the closer the spin-mixed states, the more probable the NAE. It must be remembered that the NAC is now different from zero only because of the mixing of the original singlet and triplet states caused by their SOC. The equation coupling the two spin-mixed states SM_0_ and SM_1_, resulting from the mixing of the original spin-pure states S_0_ and T_1_, will be as follows.

6 $$i{\mathrm{\psi ̇}}^{SM_{1}}\left(R,t\right)=\left({\mathrm{T̂}}_{R}+V^{SM_{1}}\left(R\right)\right)\psi^{SM_{1}}\left(R,t\right)+{\mathrm{\Lambda ̂}}^{SM_{0}SM_{1}}\psi^{SM_{0}}\left(R,t\right)$$

Spin-pure states and spin-mixed states are two complete basis sets spanning the same space, so the description of Ir3-TMCs using either representation must be equivalent. Let us first describe [Ir­(ppy)_2_(bpy)]^+^ using spin-mixed states. At the MECP, the energy separation between the latter states can be estimated as twice the SOC between the corresponding spin-pure states. The computed SOC (Section S5) between the ^3^MC_ax_ and S_0_ states at the (^3^MC_ax_)_min_ structure is 3400 cm^–1^ (0.42 eV), and for the ^3^MC_eq_ and S_0_ states at (^3^MC_eq_)_min_, it is 1676 cm^–1^ (0.21 eV). At the corresponding ^3^MC_ax_/S_0_ and ^3^MC_eq_/S_0_ MECPs, the splittings of the original spin-pure ^3^MC and S_0_ states when including the SOC are then 0.84 and 0.42 eV, respectively.

It is fundamental to understand that, depending on the relative position of the initial and final minima with respect to MECP, the SOC-induced splitting of the ^3^MC and S_0_ states has a completely different effect on the decay process. When the initial and final minima are located in opposite directions relative to MECP (i.e., when it is possible to define a reaction coordinate along which the reactant minimum, the MECP, and the product minimum are consecutive points), the MECP has a peaked topography.^19–21^ This case has been the subject of different studies.^28,35–37^ The mixing induced by the SOC results in two separated spin-adiabatic states, and what in the spin-pure picture was described as an ISC (Figure 2a, upper panel) now instead corresponds to an adiabatic process, in which the system evolves from one minimum to another along the same spin-adiabatic PES via the TS barrier (Figure 2a, bottom panel). When instead the initial and final minima are located in the same directions relative to the MECP, the MECP has a sloped topography.^19–21^ This case^38–41^ is the one for the T_1_-to-S_0_ decay mediated by ^3^MC states in Ir3-TMCs. The mixing induced by the SOC again results in two separated spin-adiabatic states, but what in the spin-pure picture was described as an ISC now still corresponds to an NAE (Figure 2b). Such an NAE is driven by the NAC between the spin-mixed states that at the original MECP are separated by a value equal to 2 times their SOC. Consequently, it is an NAE whose probability has no reason to be equal to unity at MECP, in turn meaning that the probability of reaching the S_0_ minimum has no reason to be high. In our example, according to the computed SOCs, at the ^3^MC_ax_/S_0_ and ^3^MC_eq_/S_0_ MECPs, the spin-mixed states are separated by as much as 0.84 and 0.42 eV, respectively.

**Figure 2:**
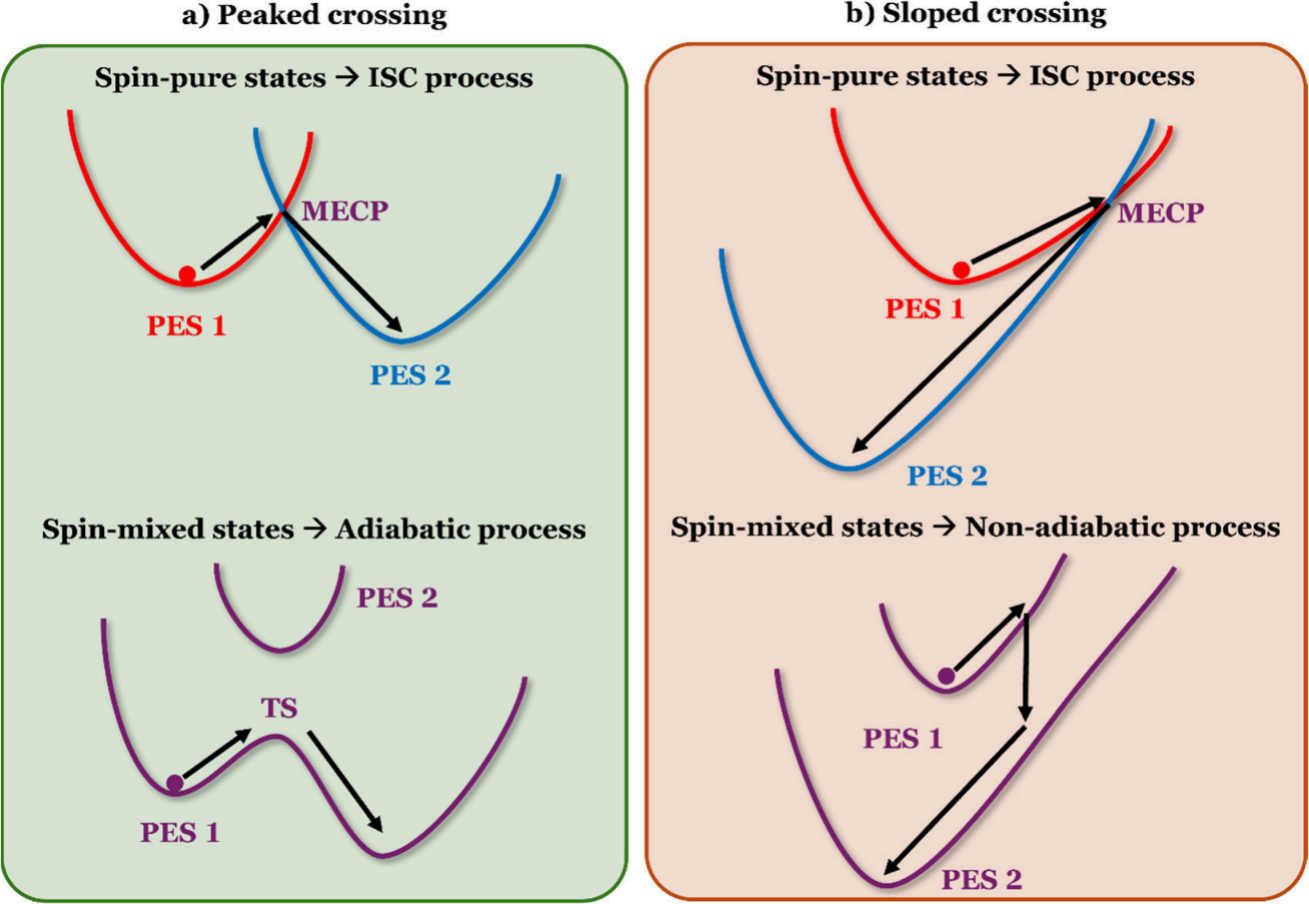
Spin-pure and spin-mixed representations of (a) peaked and (b) sloped MECP-mediated processes.

The same conclusion is also reached by using the spin-pure representation (Figure 2b, top panel). Once the trajectory propagating on PES1 reaches the MECP, the large SOC results in a high (close to unity) probability of an NAE, but due to the direction of propagation, it is the high-energy branch of PES2 that becomes populated. Once the system runs out of kinetic energy, it goes back to the MECP, where again the large SOC implies a high probability of NAE, now to the low-energy branch of PES1. This results in a very slow population decay to the low-energy branch of PES2, meaning a weak tendency to repopulate the S_0_ Franck–Condon region. This conclusion is confirmed by computing the corresponding probability and nonradiative decay rate constant, *k*_nr_(*T*), at the ^3^MC_ax_/S_0_ MECP and ^3^MC_eq_/S_0_ MECP of [Ir­(ppy)_2_(bpy)]^+^ using the nonadiabatic statistical theory (NAST) (Section S6).^28,42^ Employing the Landau–Zener equation (eqs S22 and S23), the *k*_nr_(*T*) values for the nonradiative decay path associated with the ^3^MC_ax_/S_0_ and ^3^MC_eq_/S_0_ MECPs are 1.81 × 10^–7^ and 2.32 × 10^–2^ s^–1^, respectively. Both values are significantly lower than those predicted by transition state theory (5.37 and 5.50 × 10^2^ s^–1^, respectively) and lower than the experimental total nonradiative rate constant (around 10^6^ s^–1^).^18^ Moreover, the *k*_nr_(*T*) associated with the ^3^MC_ax_/S_0_ is 5 orders of magnitude lower than the rate constant for the ^3^MC_eq_/S_0_ MECP. We hypothesize that this difference can be related to the slightly higher energy barrier to reach the ^3^MC_ax_/S_0_ MECP than the ^3^MC_eq_/S_0_ MECP (Figure S3), but also to the larger SOC at the ^3^MC_ax_/S_0_ MECP (3400 cm^–1^) than at the ^3^MC_eq_/S_0_ MECP (1676 cm^–1^). In fact, for the characterized sloped MECPs, a stronger SOC results in a larger energy gap between the spin-adiabatic PESs, leading to a less efficient nonradiative decay. To account for quantum tunneling and the nonlinear behavior of the reaction path, neglected in the Landau–Zener treatment, we computed the *k*_nr_(*T*) using the Zhu–Nakamura (ZN) transition probability equation for the nonradiative decay path mediated by the ^3^MC_ax_/S_0_ MECP.^40^ The rate calculated with the ZN transition (2.02 × 10^4^ s^–1^) indicates a massive effect of quantum tunneling on the overall nonradiative decay rate from the T_1_ state, due to the low MECP barrier and small reduced mass along the reaction coordinate (see Section S6). It is worth noticing that the ZN rate constant, in principle more accurate that the LZ result, does not describe only the probability of nonradiative decay through the MECP (since it includes tunneling). Consequently, the ZN value cannot be taken as a direct measure of the nonradiative decay rate through the MECP, which is the main subject of the present work.

An agreement between the nonradiative decay constant associated with the population of ^3^MC states and the experimental nonradiative decay constant would constitute an important piece of evidence for the model presented here in which the probability of nonradiative repopulation of the ground state is much lower than unity. Two problems must be faced in order to perform such a test. First, an Ir3-TMC where the ^3^MC-mediated decay is the main nonradiative decay path must be selected. This is because experimentally only the total nonradiative decay constant can be obtained, so only in such a case is the comparison between the theoretical and experimental values justified. In that sense, the reference [Ir­(ppy)_2_(bpy)]^+^ complex is not a good candidate, since the T_1_-to-S_0_ ISC from the emitting T_1_ minimum is probably the most important nonradiative decay path. Second, the test would be valid only if a very accurate energy barrier associated with the process can be provided. Rate constants computed using Arrhenius-like expressions (as in both TST and NAST) are extremely sensitive to the employed energy barrier, where an error as small as 0.1 eV can result in errors of orders of magnitude in the corresponding rates. We are currently evaluating the possibility of performing very computationally intensive RASPT2 calculations on specific Ir3-TMCs to obtain accurate energy barriers and nonradiative decay constants. In the present work, a first RASSCF calculation was performed on the PBE0-optimized ^3^MC_ax_/S_0_ MECP, taking as a reference the work of Bokarev et al.,^43^ followed by the computation of the corresponding spin-mixed states using the RASSI code of OpenMolcas (see Section S7).^44,45^ From such a calculation, we obtained an SOC between the ^3^MC_ax_ and S_0_ states of 3028 cm^–1^ (0.37 eV), in agreement with the TDDFT value of 3400 cm^–1^ (0.42 eV), and a splitting of the spin-mixed states of 0.76 eV, indeed equal to roughly 2 times the SOC value.

Now returning to the spin-mixed representation, one may expect that there are, however, CIs involving the two spin-mixed PESs, where the probability of an NAE is very large. Nevertheless, assuming that the SOC between the ^3^MC and S_0_ states is non-zero, no CIs between these spin-mixed states exist in the S_0_ and T_1_ two-state model.^31,46–48^ To understand this, let us recall the condition for having a CI between two pure-spin diabatic states with the same spin. In such a framework (eqs S24 and S25 of Section S8) a CI is encountered when the two states have the same energy (*H*_el_^00^ = *H*_el_^11^) and their coupling is zero (*H*_el_^01^ = 0). In our case, the two spin-pure states S_0_ and ^3^MC are our spin-diabatic states, while the corresponding spin-mixed states are the adiabatic states whose CI we are looking for. However, while the first condition (*E*(S_0_) = *E*(^3^MC)) can be satisfied (as at the MECP), the condition *H*_el_^01^ = 0 (i.e., $H_{SOC}^{{S_{0}}^{3}MC}=0$ ) is never satisfied, because the S_0_ and ^3^MC states are characterized by a large SOC.

To date, we have ignored the zero-field splitting of the T_1_ state into its three sublevels, i.e. T_–1_, T_0_, and T_1_.^49^ In a recent work, Wang and Yarkony mathematically describe the transformation from spin-pure to spin-mixed states when considering a model with the S_0_ state and the three sublevels composing the T_1_ state.^31^ They showed that two of the three sublevels have the same energy as the original T_1_ state, the energy of the third increases by a value equal to the SOC, and the energy of the spin-mixed S_0_ state decreases by a value equal to the SOC (see eqs 7 of ref 31). Only the last two spin-mixed states are interacting, which again are energetically separated by a value 2 times their original SOC.

The absence of the CIs does not mean that ^3^MC states are not involved in the nonradiative decay. ^3^MC states can still mediate a nonradiative decay in Ir3-TMCs, but according to the presented model, not through a CI, so not in a way as efficient as in CI-mediated NAEs. When ^3^MC states are accessible and significantly lower in energy than any emitting state, they will play a role, as in the related Ru­(II) d^6^ complexes [Ru­(m-bpy)_3_]^2+^ and [Ru­(tm-bpy)_3_]^2+^, where their involvement was experimentally proven.^50^ We propose that the relevance of a ^3^MC state is determined by how long Ir3-TMCs remain trapped on the ^3^MC PES (i.e., in the corresponding ^3^MC minimum). From the ^3^MC minimum, the decay back to S_0_ through MECP will have a low probability, as shown by our NA-TST calculation (see Section S6). However, if the excited state population remains trapped in the ^3^MC minimum, the small energy gap with the ground state will efficiently promote a T_1_-to-S_0_ ISC process, as in the emitting T_1_ minima part of the population decay through a T_1_-to-S_0_ ISC process whose rate constant is normally indicated as *k*_ISC_.^10,17^ The described small ^3^MC-S_0_ energy separation is caused by the broken coordination bond characterizing the ^3^MC minima, in turn leading to a massive increase in the S_0_ energy. In our example, i.e., [Ir­(ppy)_2_(bpy)]^+^, the ^3^MC-S_0_ gaps at the (^3^MC_ax_)_min_ and (^3^MC_eq_)_min_ structures are 0.55 and 0.65 eV, respectively. A key difference between the T_1_-to-S_0_ ISC process operating at the T_1_ emitting minima and that at the ^3^MC equilibrium structures can be predicted. In the former case, the T_1_ and S_0_ minima are normally nested states and the process will follow the so-called energy gap law, so as the energy separation increases, the T_1_-to-S_0_ ISC transition probability decreases. In the latter case, the large geometrical difference between the S_0_ and ^3^MC minima can instead lead to the opposite behavior, so as the energy separation increases, the T_1_-to-S_0_ ISC transition probability increases.^34,51^ Further studies are required to describe the phenomena.

The scenario exemplified for [Ir­(ppy)_2_(bpy)]^+^ is common to any Ir3-TMC, or at least to any Ir3-TMC in which ^3^MC/S_0_ MECPs display similarly large SOC (1500 cm^–1^), as is generally the case (see Section S9). Sloped ^3^MC/S_0_ MECPs are indeed present in any Ir3-TMC, as proven in Section S2. Actually, any octahedral low-spin d^6^ TMC would display such sloped ^3^MC/S_0_ MECPs, so in the case of a large SOC between the involved ^3^MC and S_0_ states, again the same conclusions here exemplified for [Ir­(ppy)_2_(bpy)]^+^ will be valid. This could be the case for Ru­(II) TMCs, whose nonradiative decay mediated by ^3^MC states is normally described in a similar way as for Ir­(III) complexes,^52–56^ and whose ^3^MC/S_0_ MECPs usually display SOC values on the order of 1000 cm^–1^.

In summary, we show that the role played by ^3^MC states in the nonradiative deactivation of Ir3-TMCs deserves reconsideration. Specifically, we showed that it is incorrect to assume that at the ^3^MC/S_0_ MECP the probability of decay back to the ground state minimum is equal to unity, and that, counterintuitively, the larger the SOC between ^3^MC and S_0_ states, the lower such a probability. Moreover, we showed that, assuming a non-zero SOC between ^3^MC and S_0_ states for all geometries, the spin-mixed states resulting from the spin-pure ^3^MC and S_0_ states do not form CIs. Consequently, even if the established strategy of avoiding ^3^MC population in order to minimize the nonradiative decay can still be valid, we postulate that the role of ^3^MC states in the nonradiative decay of Ir3-TMCs is not directly related to the ability of reaching the ^3^MC/S_0_ MECPs from the emitting minimum. Instead, we propose that the role of ^3^MC states is dictated by the tendency of Ir3-TMCs to remain trapped in the ^3^MC minima, from where, given sufficient time, the nonradiative decay to the ground state will occur by means of a T_1_-to-S_0_ ISC process. We can then suggest that in order to minimize the nonradiative decay mediated by ^3^MC states, a key design strategy will be to avoid complexes that in their ^3^MC minima display stabilizing interactions not present in the emitting structure. Such interactions could in fact determine a sizable barrier from the ^3^MC to the T_1_ minimum and in turn the trapping of the population in the ^3^MC state.

## Supplementary Material





## Notes

Graphic showing the relevance of Ir­(III) complexes for electroluminescent devices and photodynamic therapy (Section S1); properties of triplet metal-centered states (^3^MC) of Ir­(III) and Ru­(II) d^6^ transition metals under octahedral geometry (Section S2); DFT (PBE0 and B3LYP) energies, geometries, orbitals, and NTOs that characterized the nonradiative decay through ^3^MC_ax_ and ^3^MC_eq_ states for complex [Ir­(ppy)_2_(bpy)]^+^ (Section S3); derivation of the equations that govern NAEs working with spin-pure states and spin-mixed states (Section S4); computational details to obtain the SOC values at the SOC-TDDFT level of theory for complex [Ir­(ppy)_2_(bpy)]^+^ (Section S5); nonradiative decay constants computed using Landau–Zener (LZ) and Zhu–Nakamura (ZN) approximations with the NAST code (Section S6); SOC benchmark at the ^3^MC_ax_/S_0_ MECP of complex [Ir­(ppy)_2_(bpy)]^+^ computed at the RASSCF (4,2;12,9;4,2)/ANO-RCC-VDZP level of theory (Section S7); required conditions for having a CI in the framework of adiabatic and diabatic states (Section S8); and SOC-TDDFT (PBE0 and B3LYP) values for several Ir­(III) and Ru­(II) ^3^MC minima (Section S9) (PDF)
Transparent Peer Review report available (PDF)

## #

Department of Chemistry, New York University, New York, NY 10003
