# Supplementary material for: On the Role of Spin–Orbit Coupling and State-Crossing Topography in the Nonradiative Decay of Ir(III) Complexes
Source: J Phys Chem Lett. 2025 Aug 25;16(35):9004–10. doi: 10.1021/acs.jpclett.5c01776 (PMC12415876; doi:10.1021/acs.jpclett.5c01776)
Supplement: Supplementary file 1 [file jz5c01776_si_001.pdf]

Supporting Information
(Total of 22 pages)
for

The role of spin-orbit coupling and state-crossing topography in the non-radiative decay of Ir(III) complexes

Iván Soriano-Díaz,[1] Ilya D. Dergachev,[2] Sergey A. Varganov,[3] Enrique Ortí,[1] and Angelo Giussani[1,*]

[1] *Institute for Molecular Science (ICMol), Universitat de València, Catedrático José Beltrán 2, 46980 Paterna, España.*

[2] *Department of Chemistry, University of Nevada, Reno, 1664 N. Virginia Street, Reno, NV 89557-0216, USA;*
Current address: Department of Chemistry, New York University, New York, New York 10003, USA

[3] *Department of Chemistry, University of Nevada, Reno, 1664 N. Virginia Street, Reno, NV 89557-0216, USA*

Email: angelo.giussani@uv.es

Section S1. Relevance of Ir(III) complexes in the field of electroluminescent devices and photodynamic therapy

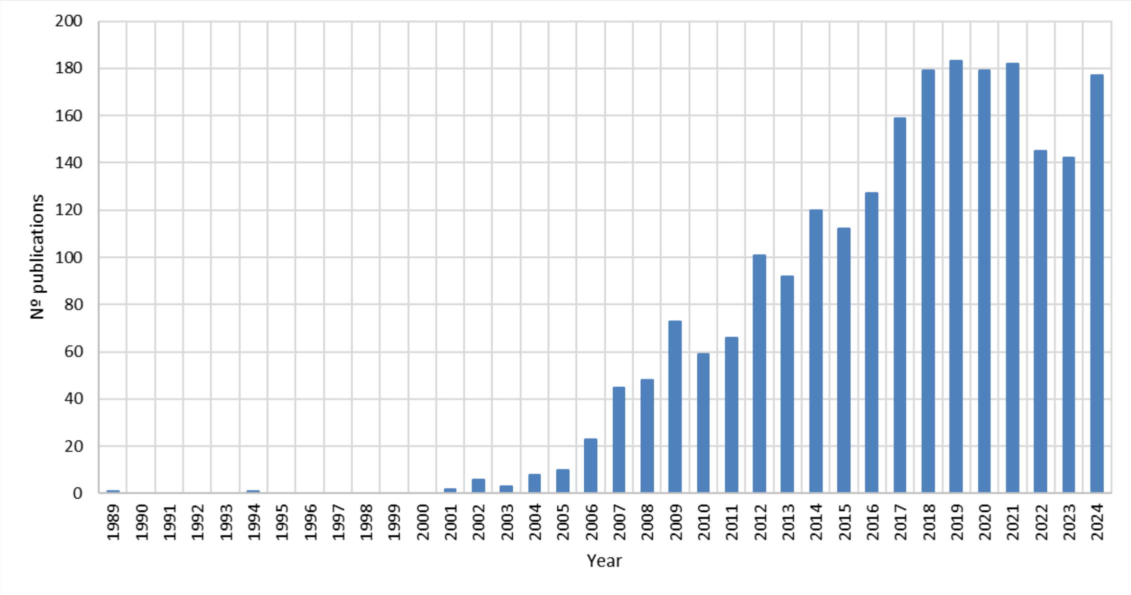

Figure S1: Number of publications per year on Web of Science studying electroluminescence applications (LECs or OLEDs) or photodynamic therapy (PDT) based on Ir(III) complexes. Search performed using TS=("Ir* III" OR "Ir(III)" OR "Ir (III)")) AND TS=("LECs" OR "LEECs" OR "electroluminescent application*" OR "photodynamic therapy" OR "OLEDs") as Booleans at 10/04/2025.

Section S2. Triplet metal-centered states of Ir(III) complexes

The Ir(III) metal centers possess electronic configurations of $5d^6$. In an octahedral coordination environment, the five *d* orbitals split into two groups according to ligand field theory: the lower-energy t_{2g} (d_{xy}, d_{xz}, and d_{yz}) orbitals and the higher-energy e_g* ($d_{x^2-y^2}$ and d_{z^2}) orbitals. According to this theory, the t_{2g} orbitals are stabilized by (2/5)Δ_o while the e_g* orbitals are destabilized by (3/5)Δ_o (see Figure S2a), where Δ_o represents the octahedral ligand field splitting energy.[1,2]

In principle, for a d^6 metal center, two electronic configurations are possible. In the low-spin configuration, all six electrons populate the t_{2g} orbitals (t_{2g}^6), whereas in the high-spin configuration the electrons occupy both ($t_{2g}^4e_g^{*2}$). The actual ground state is determined by the magnitude of Δ_o relative to the electron pairing energy. However, in Ir(III) complexes this is high enough to favors low-spin configurations and leads to a unique singlet ground state, S_0. Eventhough, the choice of ligands, as reflected in the spectrochemical series, modify the magnitude Δ_o.

According to molecular orbital theory, the e_g* orbitals from the metal center will combine with the σ orbitals of the ligands confering them an antibonding character (see Figure S2b). Consequently, metal-centered (^{3}MC) excited states of Ir(III) complexes imply an electron promotion into an e_g* orbital, which involves antibonding interactions between the metal center and the ligands. This causes that the ^{3}MC minima display a dissociated coordination bond, which is instead present in both the ground state and the emitting minima, and in turn determines that, as explained in the main text, the ^{3}MC/S_0 MECPs of Ir(III) complexes have a sloped topology.

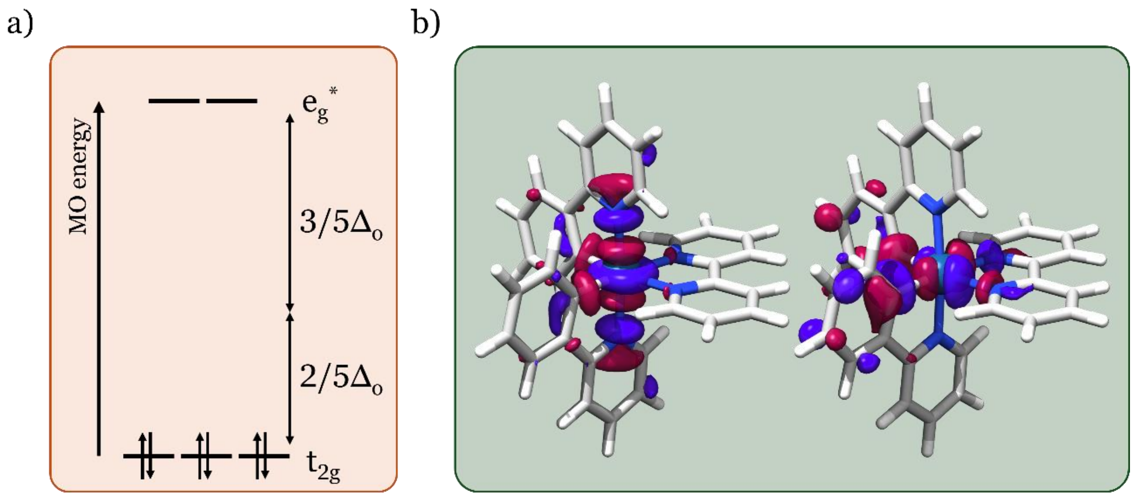

Figure S2: a) Schematic diagram showing the electronic configuration of *d*^{6} octahedral metal complexes such as Ir(III) and Ru(II) complexes. b) e_g* antibonding molecular orbitals for $[Ir(ppy)_2bpy]^+$.

An analogous discussion is valid also for Ru(II) complexes, which, as Ir(III) complexes, are low-spin d^6 octahedral complexes.

Section S3. DFT B3LYP and PBE0 energies, geometries and main orbitals characterizing the $^3MC_{ax}$ and $^3MC_{eq}$ decay paths of $[Ir(ppy)_2bpy]^+$.

Table S1: Relatives energies (in eV) for the S_0 and T_1 potential energy surfaces (PES) at (B3LYP and PBE0)/def2-SVP CPCM (CH_2Cl_2) of $[Ir(ppy)_2bpy]^+$. All the reported energies are in eV with respect to the S_0 minimum $(S_0)_{min}$ geometry.

	Geometry	S_0	T_1
B3LYP	$(S_0)_{min}$	0.00	2.59
	$(^3MLCT)_{min}$	0.27	2.34
	$(^3MLCT/^3MC_{ax\,1})_{ts}$	1.35	3.10
	$(^3MC_{ax\,1})_{min}$	2.43	2.95
	$(^3MC_{ax\,1}/S_0)_{stc\text{-}mecp}$	2.99	2.99
	$(^3MC_{ax\,2})_{min}$	1.52	3.01
	$(^3MC_{ax\,2}/S_0)_{stc\text{-}mecp}$	3.28	3.28
	$(^3MLCT/^3MC_{eq\,1})_{ts}$	2.40	2.95
	$(^3MC_{eq\,1})_{min}$	2.44	2.95
	$(^3MC_{eq\,1}/S_0)_{stc\text{-}mecp}$	2.96	2.97
PBE0	$(S_0)_{min}$	0.00	2.63
	$(^3MLCT)_{min}$	0.28	2.37
	$(^3MLCT/^3MC_{ax\,1})_{ts}$	1.55	3.26
	$(^3MC_{ax\,1})_{min}$	2.59	3.13
	$(^3MC_{ax\,1}/S_0)_{stc\text{-}mecp}$	3.17	3.17
	$(^3MLCT/^3MC_{eq\,1})_{CI\text{-}NEB}$	2.23	3.19
	$(^3MC_{eq\,1})_{min}$	2.50	3.15
	$(^3MC_{eq\,1}/S_0)_{stc\text{-}mecp}$	3.16	3.16

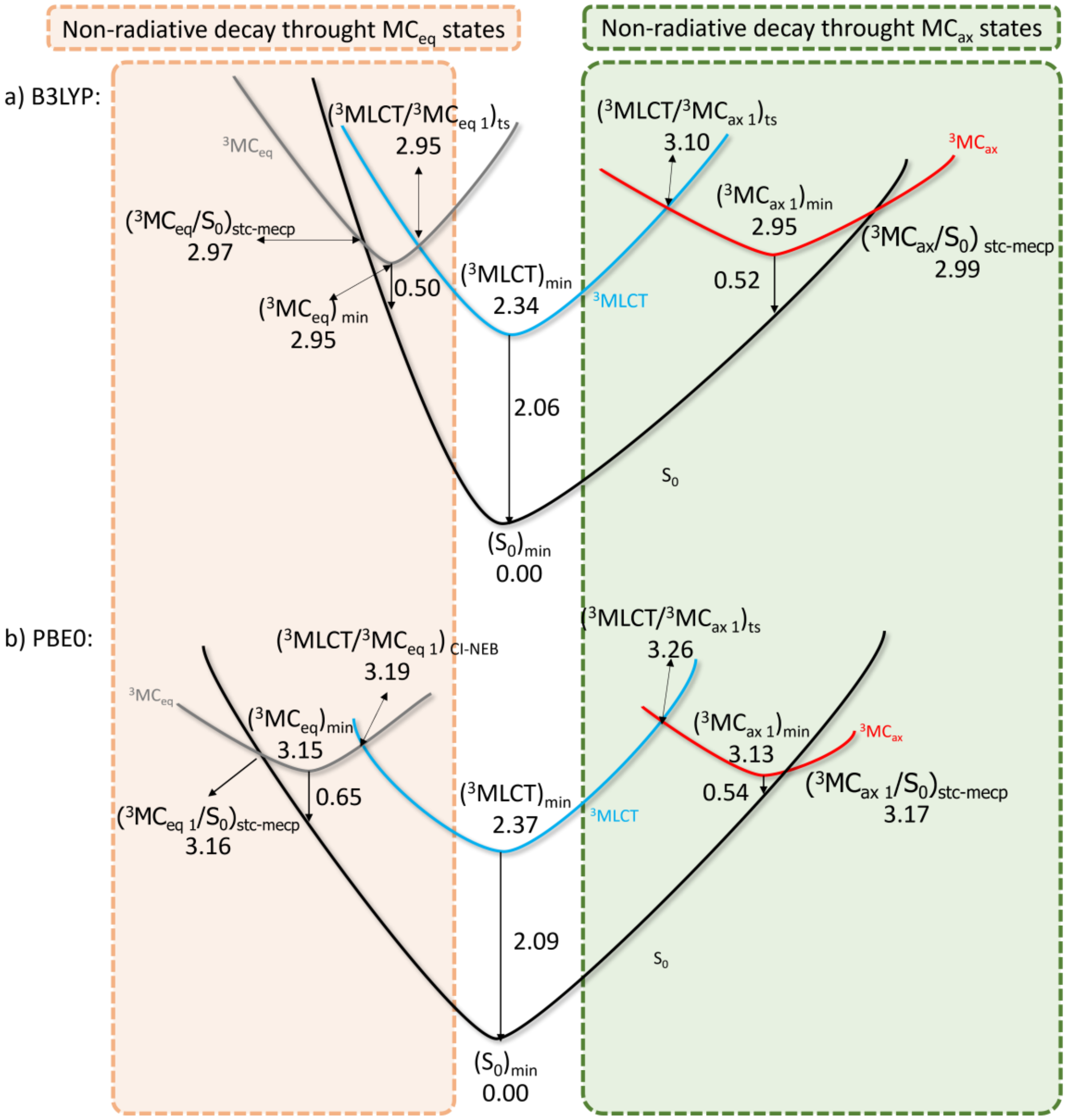

Figure S3: Schematic representation of the ^{3}MC mediated non-radiative decays of $[Ir(ppy)_2bpy]^+$ computed at B3LYP/def2-SVP CPCM (CH_2Cl_2) (a) and PBE0/def2-SVP CPCM (CH_2Cl_2) (b). The left side (in orange box) shows the non-radiative decay path through the MC_{eq} states. The right side (in green box) shows the non-radiative decay path through the MC_{ax} states. All the reported energies are in eV with respect to the S_0 minimum $(S_0)_{min}$ geometry.

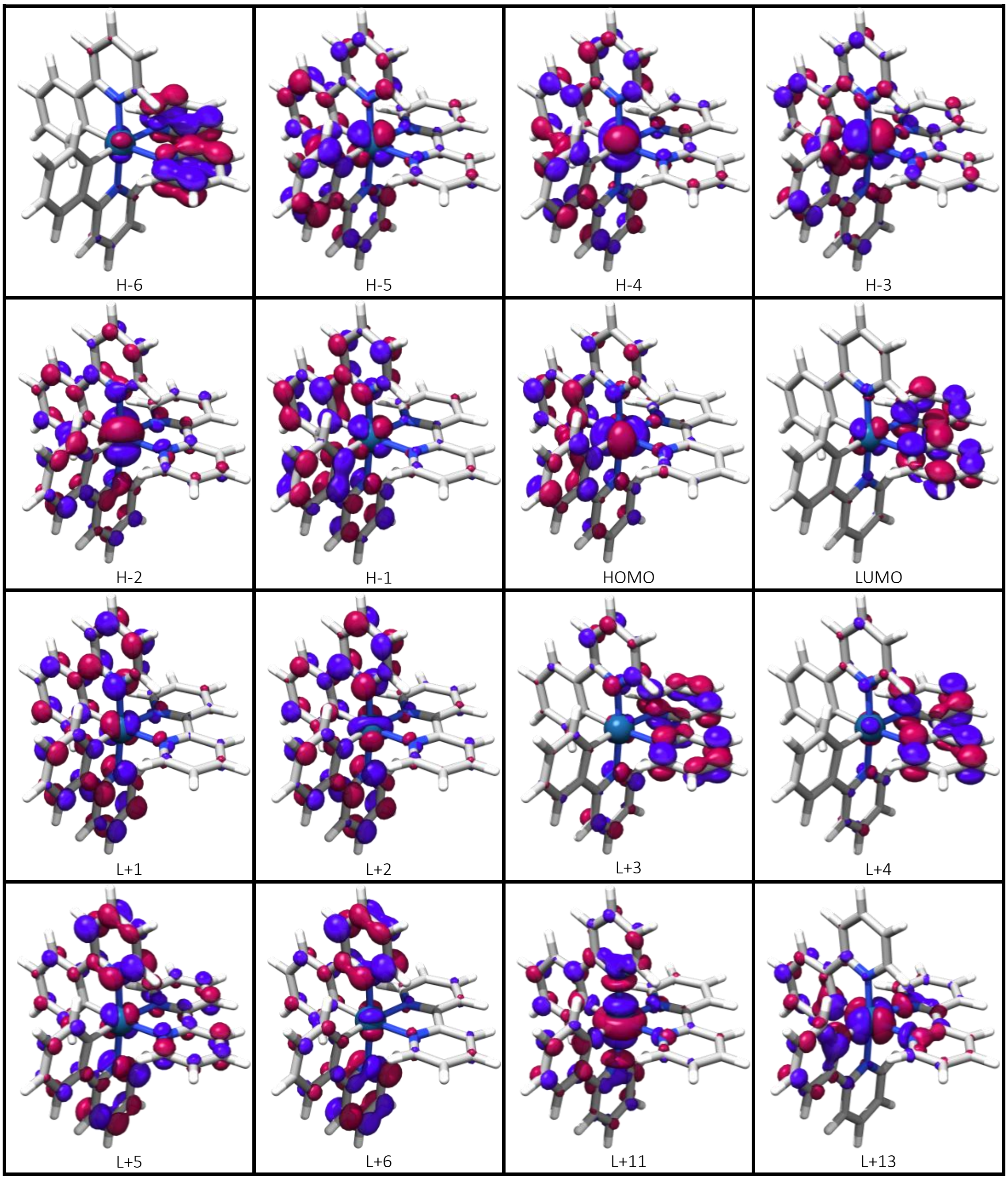

Figure S4: Molecular orbitals isosurfaces contour values (± 0.04 a.u.) of $[Ir(ppy)_2bpy]^+$ computed at $(S_0)_{min}$ geometry at PBE0/def2-SVP CPCM (CH_2Cl_2) level of theory.

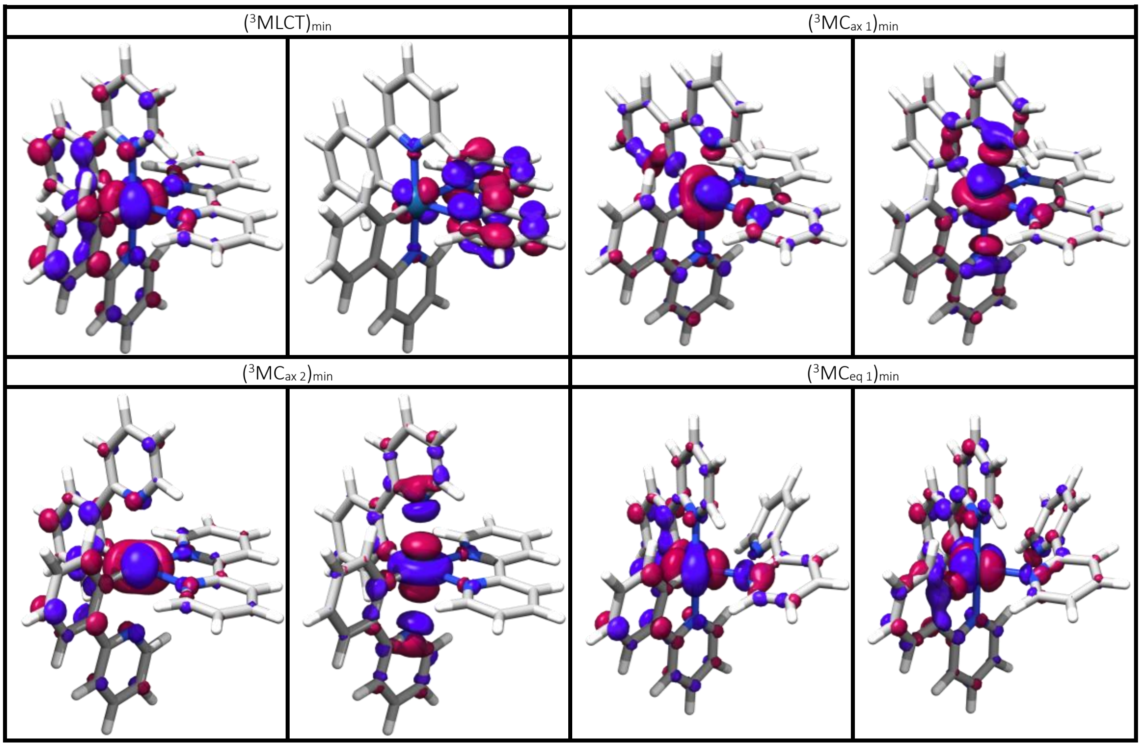

Figure S5: Natural transition orbitals (NTOs) isosurfaces contour values (± 0.04 a.u.) of $[Ir(ppy)_2bpy]^+$ that characterized MLCT, MC$_{ax\ 1}$, MC$_{ax\ 2}$, and MC$_{eq\ 1}$ triplet minima computed at at B3LYP/def2-SVP CPCM (CH_2Cl_2) level of theory.

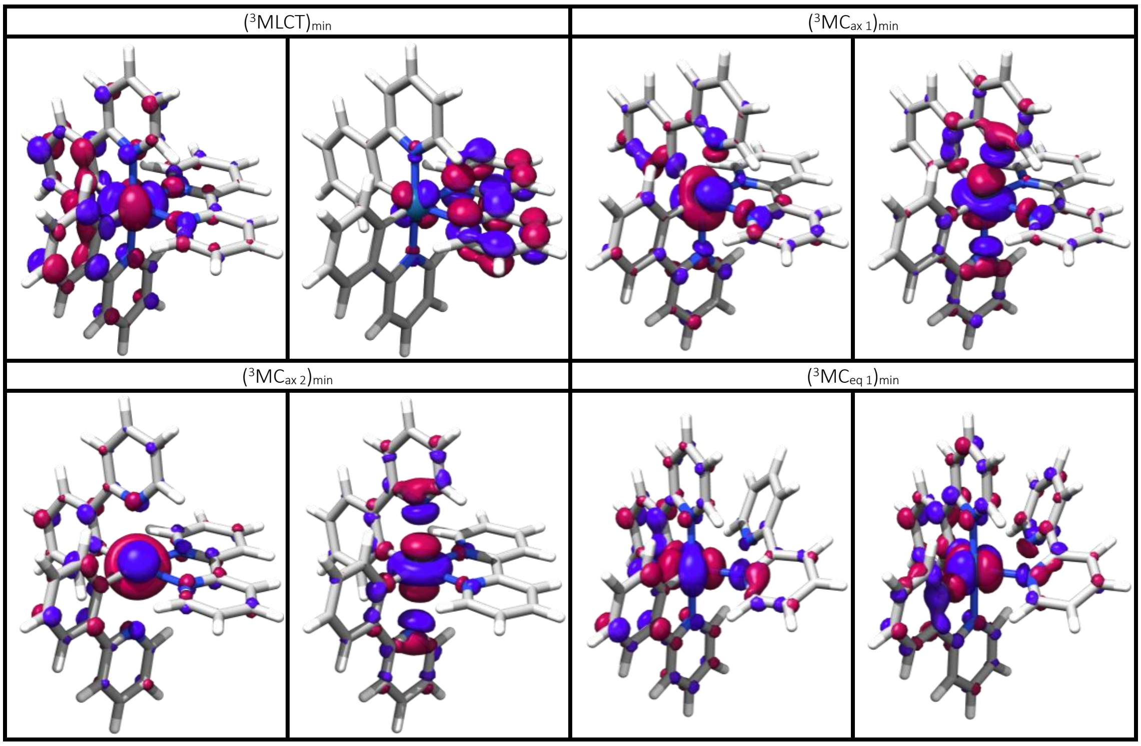

Figure S6: Natural transition orbitals (NTOs) isosurfaces contour values (± 0.04 a.u of $[Ir(ppy)_2bpy]^+$ that characterized MLCT, MC$_{ax\ asi}$, MC$_{ax\ sim}$, and MC$_{eq}$ triplet minimacomputed at at PBE0/def2-SVP CPCM (CH_2Cl_2) level of theory.

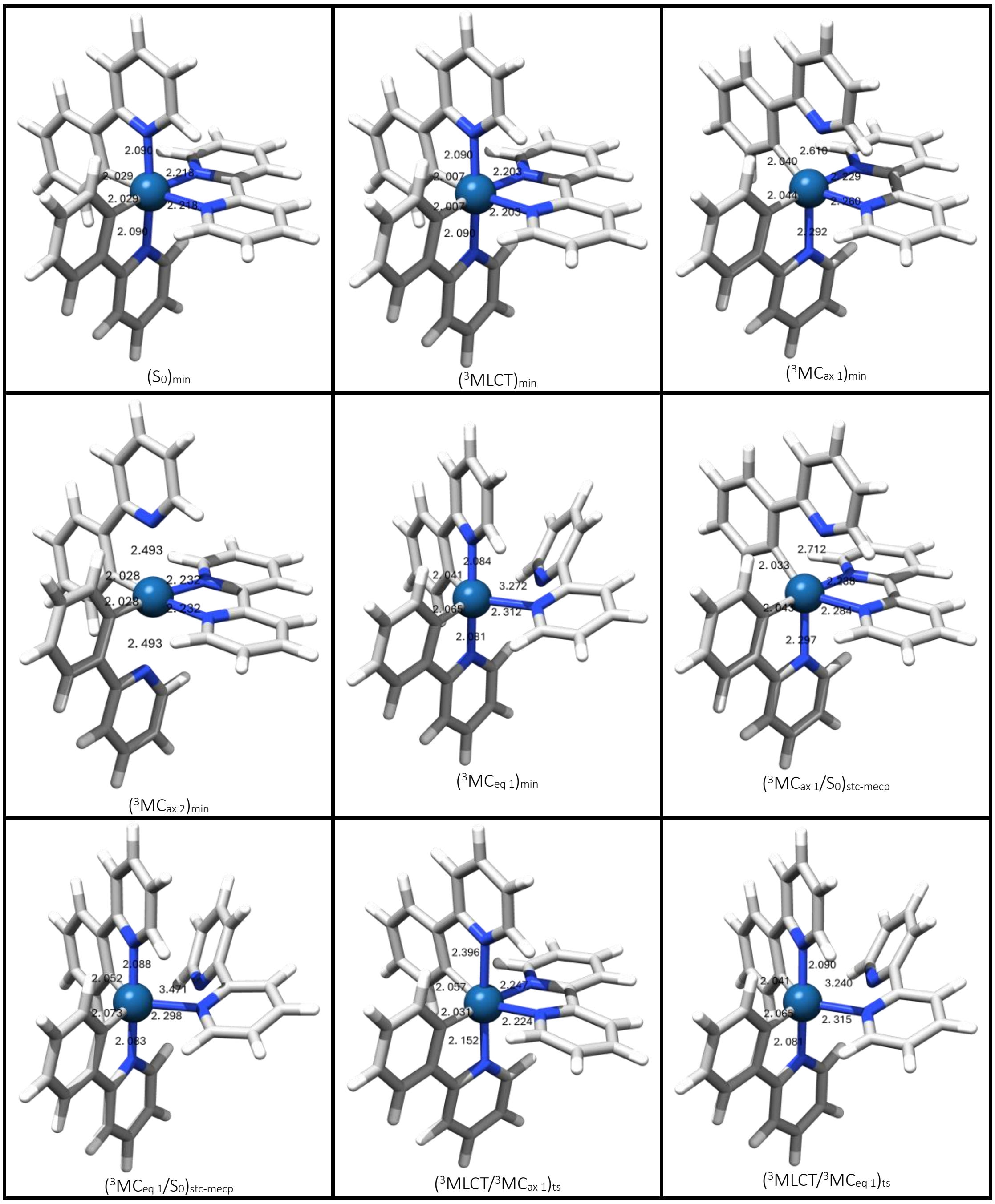

Figure S7: Main geometrical parameters for all the critical points of $[Ir(ppy)_2bpy]^+$ computed at at B3LYP/def2-SVP CPCM (CH_2Cl_2) level of theory.

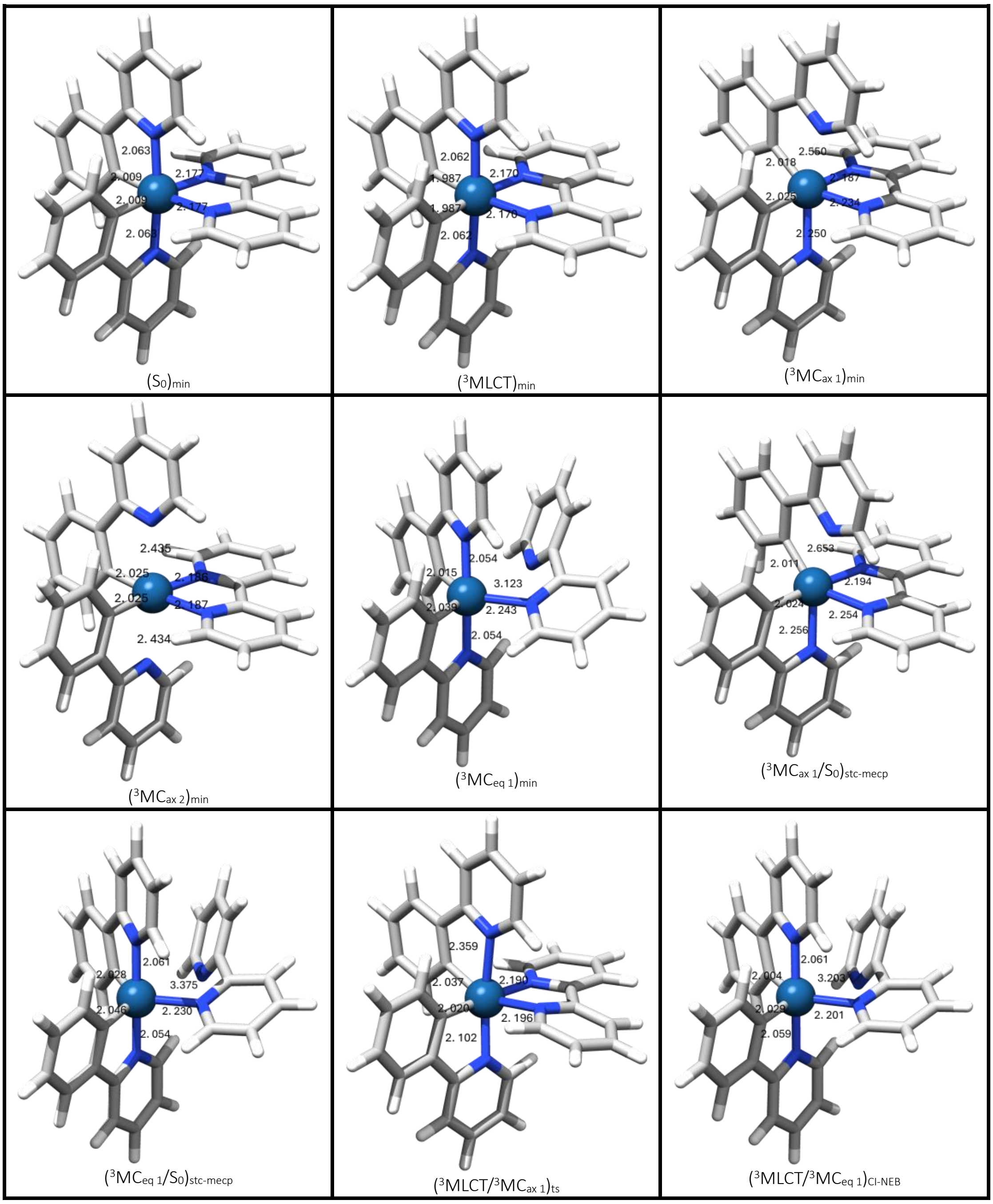

Figure S8: Main geometrical parameters for all the critical points of $[Ir(ppy)_2bpy]^+$ computed at at PBE0/def2-SVP CPCM (CH_2Cl_2) level of theory.

Section S4. Derivation of the equations that govern NAEs working with spin-pure states and spin-mixed states.

The time-dependent Schrödinger equation for a generic molecule can be written as follow:

$$i\dot{\Psi}(\boldsymbol{r},\boldsymbol{R},t) = \hat{H}_{mol}\Psi(\boldsymbol{r},\boldsymbol{R},t) \quad \text{eq. S1}$$

where $\hat{H}_{mol}$ is the molecular Hamiltonian and $\boldsymbol{r},\boldsymbol{R}$ and t correspond to the electronic, nuclear and time coordinates, respectively. $\hat{H}_{mol}$ is in turn composed of the nuclear kinetic operator $\hat{T}_R$, the electronic Hamiltonian $\hat{H}_{el}$, and the SOC term $\hat{H}_{SOC}$.

$$\hat{H}_{mol} = \hat{T}_R + \hat{H}_{el} + \hat{H}_{SOC} \quad eq.S2$$

The eigenfunctions of the electronic Hamiltonian $|s\rangle$ (i.e.: the adiabatic electronic states) form a complete basis set, so it is possible to expand the wave-function $\Psi(\boldsymbol{r},\boldsymbol{R},t)$ in such a basis set without introducing any approximation:

$$\hat{H}_{el}|s\rangle = V^s(\boldsymbol{R})|s\rangle \quad eq.S3$$

$$\Psi(\boldsymbol{r},\boldsymbol{R},t) = \sum_s \psi^s(\boldsymbol{R},t)\,|s\rangle \quad eq.S4$$

Substituting such an expression in the time-dependent Schrödinger equation S1, neglecting the $\hat{H}_{SOC}$ term, and projecting on the generic electronic state $|s'\rangle$, it is possible to obtain:

$$i\sum_s \dot{\psi}^s(\boldsymbol{R},t)\langle s'|s\rangle = \sum_s \langle s'|(\hat{T}_R+\hat{H}_{el})\psi^s(\boldsymbol{R},t)|s\rangle \quad eq.S5$$

It can be proved that:

$$\langle s'|\hat{H}_{el}\psi^s(\boldsymbol{R},t)|s\rangle = \delta_{ss'}V^{s'}(\boldsymbol{R})\psi^{s'}(\boldsymbol{R},t)|s'\rangle \quad eq.S6$$

$$\langle s'|\hat{T}_R\psi^s(\boldsymbol{R},t)|s\rangle = (\delta_{ss'}\hat{T}_R + \hat{\Lambda}^{ss'})\psi^s(\boldsymbol{R},t) \quad eq.S7$$

Where $V^{s'}(\boldsymbol{R})$ is the electronic energy of state $|s'\rangle$, and $\hat{\Lambda}^{ss'}$ are the so-called non-adiabatic couplings (NACs).[3,4]

Using these expressions, it is possible to arrive at the next equation that clearly shown that what causing that part of the population of the generic state $|s'\rangle$ to pass to another state $|s\rangle$, is the corresponding NAC ($\hat{\Lambda}^{ss'}$), which is in fact the term that in the next equation connects the time evolution of the population of state $|s'\rangle$ (i.e.: $\dot{\psi}^{s'}(\boldsymbol{R},t)$) with the population of the other state $|s\rangle$ (i.e.: $\psi^s(\boldsymbol{R},t)$):

$$i\dot{\psi}^{s'}(\boldsymbol{R},t) = \left(\hat{T}_R + V^{s'}(\boldsymbol{R})\right)\psi^{s'}(\boldsymbol{R},t) + \sum_s \hat{\Lambda}^{ss'}\,\psi^s(\boldsymbol{R},t) \quad eq.S8$$

The NACs are mostly composed of the so-called first-order non-adiabatic coupling, which, using the Hellmann-Feynman theorem can be proven to be inversely proportional to the energy separation of the two involved states:

$$\hat{\Lambda}^{ss'} \cong -\hbar^2\langle s|\hat{\nabla}_R|s'\rangle\frac{1}{M}\nabla_{\boldsymbol{R}} \quad eq.S9$$

$$\langle s|\hat{\nabla}_R|s'\rangle = \frac{\langle s|\hat{\nabla}_R\hat{H}_{el}|s'\rangle}{V^{s'}(\boldsymbol{R}) - V^s(\boldsymbol{R})} \quad eq.S10$$

In the particular case in which we expand the wave-function in the basis of only two adiabatic electronic states of pure-spin having the same spin symmetry, and for example the S_0 and S_1 states, we obtain, considering that the initial population is in the S_1 state:

$$i\dot{\psi}^{S_1}(\boldsymbol{R},t) = \left(\hat{T}_R + V^{S_1}(\boldsymbol{R})\right)\psi^{S_1}(\boldsymbol{R},t) + \hat{\Lambda}^{S_0S_1}\psi^{S_0}(\boldsymbol{R},t) \quad eq.S11$$

And the corresponding IC process will have a higher probability at regions of CI between the S_0 and S_1 states.

Let's again write the wave function on the basis of adiabatic electronic states of pure-spin, but now allowing them to have different spin symmetry. Considering the $\hat{H}_{SOC}$ term in the molecular Hamiltonian appearing in the time-dependent Schrödinger equation,[5] and working in an analogous way as before, we obtain:

$$\langle s'|\hat{H}_{el}\psi^{s}(\boldsymbol{R},t)|s\rangle = \delta_{ss'}V^{s'}(\boldsymbol{R})\psi^{s'}(\boldsymbol{R},t)|s'\rangle \quad eq.S12$$

$$\langle s'|\hat{T}_R\psi^{s}(\boldsymbol{R},t)|s\rangle = (\delta_{ss'}\hat{T}_R + \delta_{\sigma(s)\sigma(s')}\hat{\Lambda}^{ss'})\psi^{s}(\boldsymbol{R},t) \quad eq.S13$$

$$\langle s'|\hat{H}_{SOC}\psi^{s}(\boldsymbol{R},t)|s\rangle = (1-\delta_{\sigma(s)\sigma(s')})H_{SOC}^{s's}\psi^{s}(\boldsymbol{R},t) \quad eq.S14$$

Notice that in eq. S13 the NAC term appears only for state of the same spin symmetry (ensure by the presence of the $\delta_{\sigma(s)\sigma(s')}$ term, where the symbol $\sigma(s)$ indicate the spin coordinate of state $|s\rangle$), while in eq. S14 the SOC term appears only for state of different spin symmetry (ensure by the presence of the $1-\delta_{\sigma(s)\sigma(s')}$ term).

Now using these expressions and considering the specific case of just one singlet and triplet state (i.e.: the S_0 and T_1 states) we can write:

$$i\dot{\psi}^{T_1}(\boldsymbol{R},t) = \left(\hat{T}_R + V^{T_1}(\boldsymbol{R})\right)\psi^{T_1}(\boldsymbol{R},t) + H_{SOC}^{S_0T_1}\psi^{S_0}(\boldsymbol{R},t) \quad eq.S15$$

So now what connects the time evolution of the population of initially populated triplet state (i.e.: $\dot{\psi}^{T_1}(\boldsymbol{R},t)$) with the population of the ground state (i.e.: $\psi^{S_0}(\boldsymbol{R},t)$), is the $H_{SOC}^{S_0T_1}$ term.

Finally, we will use the spin-mixed states $|SM\rangle$ resulting from the diagonalization of the Hamiltonian formed by the electronic Hamiltonian plus the $\hat{H}_{SOC}$ term in order to expand the wave-function, again in the case of considering just two states:

$$\begin{pmatrix} V^{S_0} & H_{SOC}^{S_0T_1} \\ H_{SOC}^{S_0T_1} & V^{T_1} \end{pmatrix} \xrightarrow{diagonalization} \begin{pmatrix} V^{SM_0} & 0 \\ 0 & V^{SM_1} \end{pmatrix} \quad eq.S16$$

$$|SM_1\rangle = c_{S_0}^{SM_1}|S_0\rangle + c_{T_1}^{SM_1}|T_1\rangle \quad eq.S17$$

$$|SM_2\rangle = c_{S_0}^{SM_2}|S_0\rangle + c_{T_1}^{SM_2}|T_1\rangle \quad eq.S18$$

Proceeding as before

$$\langle SM'|(\hat{H}_{el} + \hat{H}_{SOC})\psi^{SM}(\boldsymbol{R},t)|SM\rangle = \delta_{SMSM'}V^{SM'}(\boldsymbol{R})\psi^{SM'}(\boldsymbol{R},t)|SM'\rangle \quad eq.S19$$

$$\langle SM'|\hat{T}_R\psi^{SM}(\boldsymbol{R},t)|SM\rangle = (\delta_{SMSM'}\hat{T}_R + \hat{\Lambda}^{SMSM'})\psi^{SM}(\boldsymbol{R},t) \quad eq.S20$$

That for the specific case of just two spin-mixed states resulting from the combination of the original S_0 and T_1 states, leads to the next equation:

$$i\dot{\psi}^{SM_1}(\boldsymbol{R},t) = \left(\hat{T}_R + V^{SM_1}(\boldsymbol{R})\right)\psi^{SM_1}(\boldsymbol{R},t) + \hat{\Lambda}^{SM_0SM_1}\psi^{SM_0}(\boldsymbol{R},t) \quad eq.S21$$

Eq. S21 clearly shows that what couple spin-mixed states then allowing for non-adiabatic events to occurs, are again the NACs, whose value again is inversely proportional to the energy separation of the two involved spin-mixed states.

Section S5. Computational details used for calculating the SOC values of [Ir(ppy)$_2$(bpy)]$^+$.

The values of the SOC have been obtained by performing SOC-TDDFT calculations with ORCA 5.04,[6] using the PBE0 functional,[7] employing the ZORA Hamiltonian[8] to simulate the relativistic effects, a mean-field spin−orbit operator,[9] and the basis-set ZORA-def2-SVP[10] for non-Ir atoms and ZORA-def2-TZVP[11] for the Ir center.

Section S6. Calculating non-radiative decay probabilities using the NAST code.

In this work, we consider a sloped intersection of two spin-diabatic states with different spin multiplicity strongly coupled by the spin-orbit coupling at MECP. These states are shown in Figure S9 (see also Figure 2b in the main text). The relaxation process starts at the minimum of the spin-diabatic potential of the excited ^{3}MC state (solid blue line) and propagates towards the MECP with the ground S_0 state potential (solid orange line). Large spin-orbit coupling (>1000 cm^{-1}) between the two states at MECP results in the population transfer to the upper branch of the spin-diabatic potential of the ground state. When the system runs out of kinetic energy and the population starts moving in the opposite direction, the system will pass through MECP a second time and a second population transfer back to the potential of the excited triplet state occurs. Thus, the probability of the population transfer to the lower branch of the spin-diabatic potential of the ground state becomes very small. This means that the probability of re-population of the ground state Franck-Condon region becomes very small. In the adiabatic representation, a large spin-orbit coupling results in a large energy gap between the two adiabatic potentials. Thus, a sloped intersection of two states of different spin multiplicities and their large coupling via spin-orbit interaction results in the population locked on the upper adiabatic potential (dashed red line), making the transition probability to the lower spin-adiabatic state (dashed green line) very small.

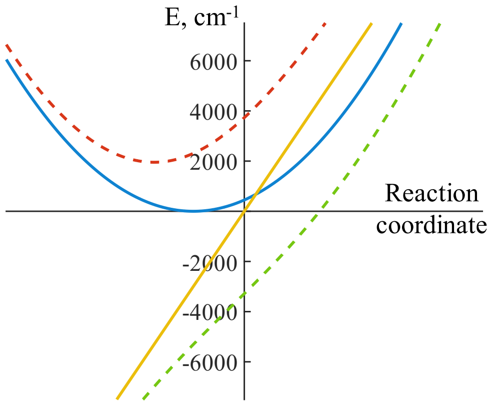

Figure S9. Sloped intersection of two spin-diabatic potentials (solid lines) and two spin-adiabatic potentials (dashed lines) after adiabatization with spin-orbit coupling.

In order to quantitatively support the conclusion that sloped MECPs characterized by large SOC do not efficiently mediate the re-population of the ground state Franck-Condon region, we calculate such a probability for the specific case of the path from the $^3MC_{ax}$ minimum to the $^3MC_{ax}/S_0$ MECP described in the main text for the $[Ir(ppy)_2(bpy)]^+$ using the NAST code.[12]

At the MECP defined by two spin-diabatic PESs, the probability of transition between the corresponding spin-adiabatic surfaces can be computed according to the following Landau-Zener equation:

$$p_{\mathrm{LZ}}(\varepsilon_\perp) = \exp\left(-\frac{2\pi H_{\mathrm{SO}}^2}{\hbar|\Delta \mathbf{g}|} \sqrt{\frac{\mu_\perp}{2(\varepsilon_\perp - E_X)}} \right) \quad eq.S22$$

where H_{SO} is the spin-orbit coupling of the two spin-diabatic PESs at MECP, $\hbar$ is the reduced Planck's constant, $|\Delta\mathbf{g}|$ is the norm of the gradient parallel to the reaction coordinate at MECP, $\mu_\perp$ is the reduced mass along the reaction coordinate at MECP, $\varepsilon_\perp$ is the reaction coordinate energy (i.e. the energy of the system along the coordinate leading to MECP, which in this specific case is an Ir-N coordination bond), and E_{X} is the MECP energy barrier with respect to the reactant minimum (in this case the $^3MC_{ax}$ minimum).
Considering the case above described, according to which the system can pass two times through MECP, the probability of decaying to the ground state Franck-Condon region (i.e. the probability of population transfer to the lower branch of the spin-diabatic potential of the ground state) can be computed with the following equation:[12]

$$P_{LZ}(\varepsilon_\perp) = 2 * [p_{\mathrm{LZ}}(\varepsilon_\perp) - p_{\mathrm{LZ}}(\varepsilon_\perp)^2] \quad eq.S23$$

Figure S10 present a plot of the probability of decaying to the ground state Franck-Condon region at MECP as a function of the reaction coordinate energy ($\varepsilon_\perp$) for the path defined by the $^3MC_{ax}$ minimum and $^3MC_{ax}/S_0$ MECP of $[Ir(ppy)_2(bpy)]^+$. It is possible to appreciate that even at the very high value of the reaction coordinate energy equal to 1 eV, corresponding to the case of having 1 eV in the coordinate connecting the $^3MC_{ax}$ and $^3MC_{ax}/S_0$ MECP structures, the probability of decay is still significantly lower than 1 (specifically, it is equal to 0.24).

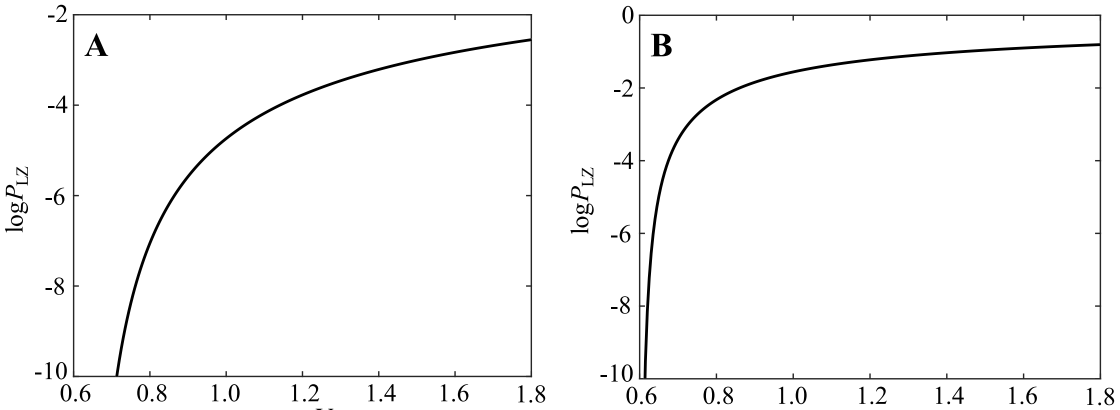

Figure S10. Probability of decaying to the ground state Franck-Condon region at MECP, computed using equation S22, as a function of the reaction coordinate energy ($\varepsilon_\perp$) for the path defined by the T_1 minimum and $^3MC_{ax}/S_0$ (A) or $3MC_{eq}/S_0$ (B) MECP of $[Ir(ppy)_2(bpy)]^+$.

To estimate the effect of quantum tunneling on the reaction rate, we compared the rate constants calculated with Landau-Zener (LZ) transition probability formula and the rate constants calculated with the Zhu-Nakamura (ZN) probability formulas as implemented in the NAST package for the $^3MC_{ax}/S_0$ MECP.[12] The calculation of the ZN rate constant requires explicit reaction potentials of the two states (Figure S5). These potentials were obtained by fitting the intrinsic reaction coordinate points calculated with ORCA 5.0.4 program package,[6] with the fourth-order polynomials. Using these potentials, the ZN rate constant was found to be $2.02 \cdot 10^4$ s^{-1} at 298 K, which can be compared to the LZ rate constant of $1.45 \cdot 10^{-7}$ s^{-1}. We attribute this large difference between the two rate constants to the effect of quantum tunneling through the barrier for a system with a relatively small reduced mass associated with the reaction coordinate of 9.1 amu predicted by NAST.

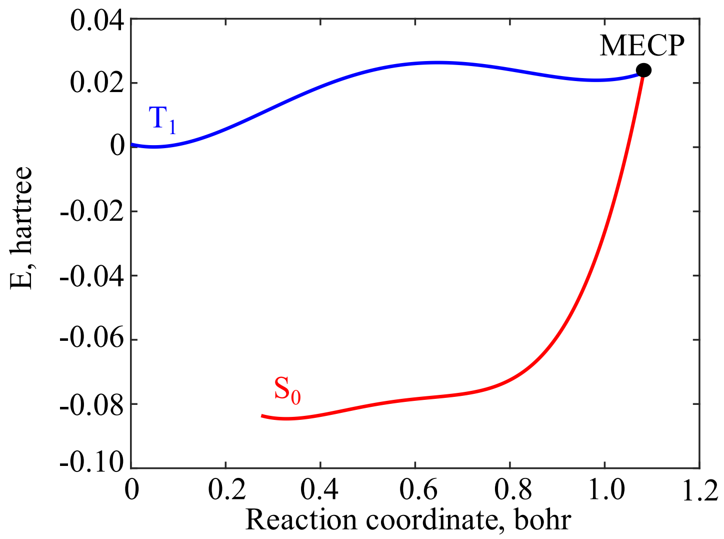

Figure S11. The fitted $^3MC_{ax}/S_0$ MECP ⟶ T_1 (blue) and $^3MC_{ax}/S_0$ MECP ⟶ S_0 (red) reaction potentials used in the Zhu-Nakamura calculations. The reaction coordinate and energy values are given relative to the T_1 minimum.

Section S7. RASSCF and RASSI-SOC calculation

To guarantee that the SOC-TDDFT calculations (see Sections S4 and S9) are accurate to support our conclusions, we performed a RASSCF (4,2;12,9;4,2) calculations with OpenMolcas software (version 24.02).[13] The Atomic Natural Orbital Relativistic Consistent Correlated (ANO-RCC- VDZP) were chosen for all the atoms. This calculation was performed at $^3MC_{ax}/S_0$ MECP computed at UPBE0/def2-SVP (CH_2Cl_2) level of theory. The active space is composed of three occupied 5d orbitals, two σ Ir-C bonds, one occupied π on the bpy ligand and two occupied π for each ppy ligand. The two anti-bonding 5d orbitals, one π^* on the bpy ligand and two π^* for each ppy ligand (See Figure SX). This active space was selected to describe the main ^3MC character of this MECP but also to include the smaller contributions such as: ^3MLCT, ^3LMCT, and ^3LLCT. The number of orbitals were based on the previous work of Bokarev, et al.[14]

The SOC were computed using RASSI module of OpenMolcas program using the first singlet and triplet states, obtaining a value of 3028 cm^{-1} , in agreement with the SOC-TDDFT result (3400 cm^{-1}). The resulting spin-mixed states are separated by 0.76 eV, almost two times the SOC value.

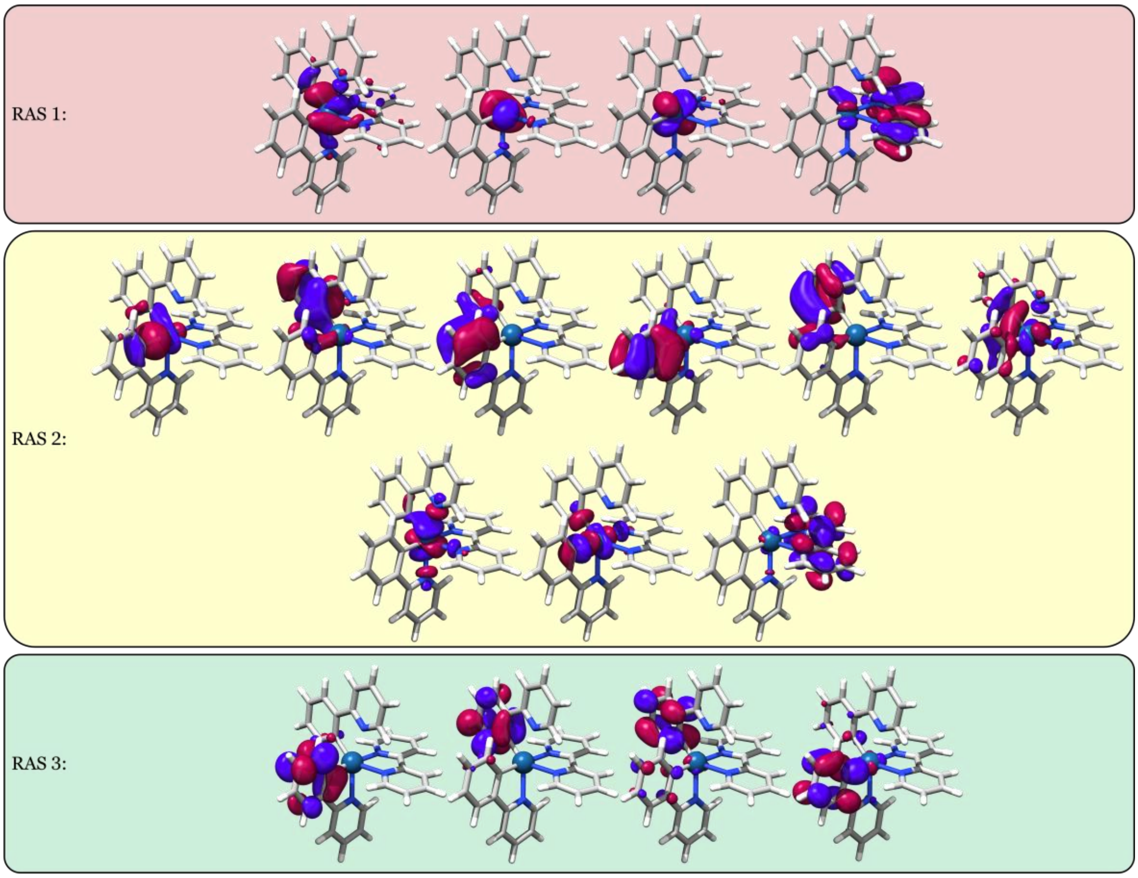

Figure S12: Employed RASSCF (4,2;12,9;4,2) active space computing 15 roots for triplets and singlets states at the $^3MC_{ax}/S_0$ MECP, in turn optimized with UPBE0/def2-SVP (CH_2Cl_2). Isosurface contour values equals to ±0.04 a.u.

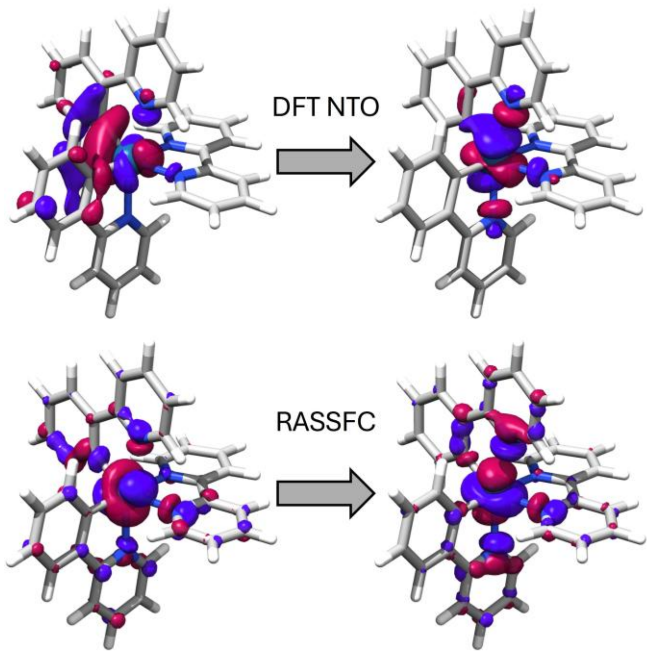

Figure S13:DFT Natural transition orbitals (NTO) and main RASSCF orbitals describing the ^{3}MC state computed with DFT and RASSCF, respectively.

Section S8. Required conditions for having a CI in the framework of adiabatic and diabatic states.

Considering two adiabatic states a^0 and a^1 of pure-spin having the same spin symmetry, by definition a CI is a point in which the two states have the same energy. In terms of the matrix elements of the electronic Hamiltonian (H_{el}^{ij}), and remembering that adiabatic states are by definition eigenfunctions of the electronic Hamiltonian, we have a CI when the following condition is fulfilled.

$$H_{el}^{a^0a^0} = H_{el}^{a^1a^1} \quad eq.S23$$

Considering instead two diabatic states d^0 and d^1 derivable form a unitary transformation of the a^0 and a^1 adiabatic states, and remembering that diabatic states are no longer eigenfunction of the electronic Hamiltonian, it is possible to prove that we have a CI when the following two conditions are fulfilled:[15]

$$H_{el}^{d^0d^0} = H_{el}^{d^1d^1} \quad eq.S24$$

$$H_{el}^{d^0d^1} = 0 \quad eq.S25$$

Two spin-pure adiabatic states of different spin symmetry (as for example a singlet state and a triplet state), obtained as eigenfunction of the spin-free electronic Hamiltonian, behave as diabatic states and in fact the corresponding PES can cross. In such a case equation 22 can be fulfilled, while, as long as there is SOC between the two, equation 23 will never be satisfied. Consequently, as long there is a non-null SOC between two spin-pure states of different spin symmetry, there will never be a CI for the corresponding spin-mixed states.

Section S9. Examples of Ir(III) and Ru(II) complexes whose ^{3}MC minima display a broken coordination bond and a large SOC with the ground state.

<table>
<tr><th colspan="12">Ir(III) Complexes</th></tr>
<tr><td colspan="12">$[Ir(ppy)_2bpy]^+$ ref. 16-17</td></tr>
<tr><td colspan="4">$reMC_{ax\,1}$</td><td colspan="4">$MC_{ax\,2}$</td><td colspan="4">$MC_{eq\,1}$</td></tr>
<tr><td colspan="4">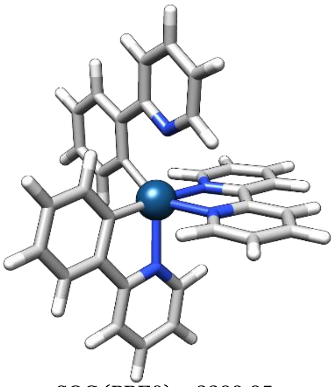
SOC (PBE0) = 3399.95
SOC (B3LYP) = 3362.43</td><td colspan="4">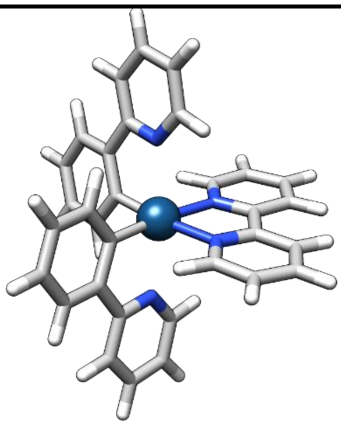
SOC (PBE0) = 3044.23
SOC (B3LYP) = 1683.64</td><td colspan="4">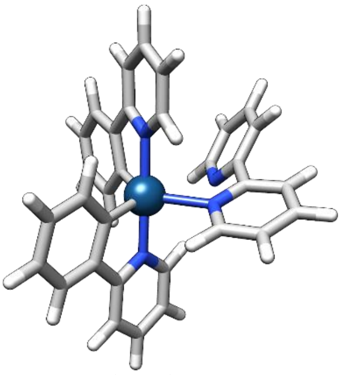
SOC (PBE0) = 1676.19
SOC (B3LYP) = 1897.39</td></tr>
<tr><td colspan="12">$[Ir(ppy)_2bpyph]^+$ ref. 16-17</td></tr>
<tr><td colspan="3">$MC_{ax\,1}$</td><td colspan="3">$MC_{ax\,2}$</td><td colspan="3">$MC_{eq\,1}$</td><td colspan="3">$MC_{eq\,2}$</td></tr>
<tr><td colspan="3">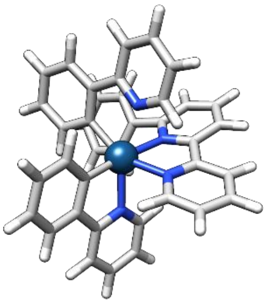
SOC (PBE0) = 3445.39
SOC (B3LYP) = 3471.38</td><td colspan="3">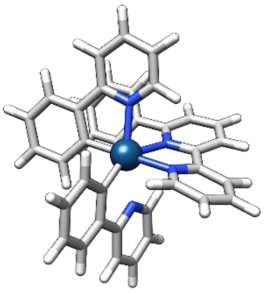
SOC (PBE0) = 3456.22
SOC (B3LYP) = 3467.86</td><td colspan="3">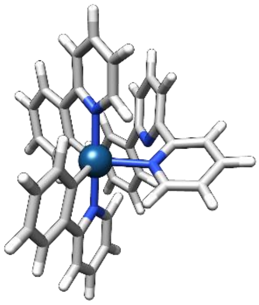
SOC (PBE0) = 1230.39
SOC (B3LYP) = 1505.32</td><td colspan="3">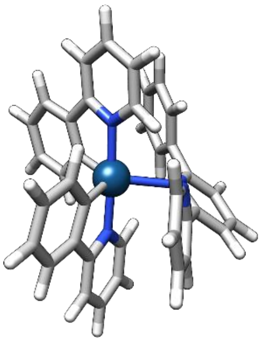
SOC (PBE0) = 1747.99
SOC (B3LYP) = 2028.45</td></tr>
<tr><td colspan="12">$[Ir(ppy)_2bpyph_2]^+$ ref. 16-17</td></tr>
<tr><td colspan="6">$MC_{ax\,1}$</td><td colspan="6">$MC_{eq\,1}$</td></tr>
<tr><td colspan="6">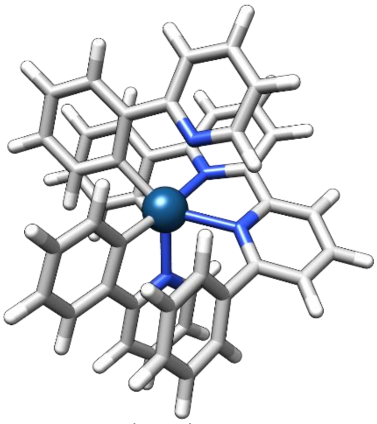
SOC (PBE0) = 2978.27
SOC (B3LYP) = 3165.88</td><td colspan="6">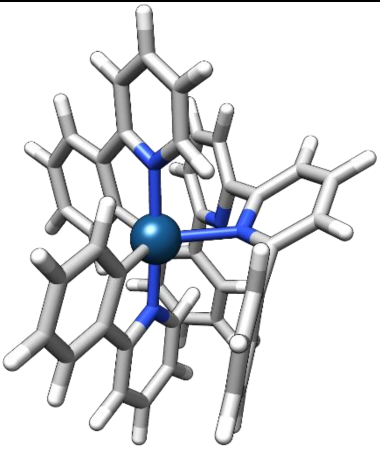
SOC (PBE0) = 1755.85
SOC (B3LYP) = 2037.63</td></tr>
</table>

[Ir(diF-ppy)$_2$dtb-bpy]$^{+}$ ref. 18		
$MC_{ax\ 1}$	$MC_{ax\ 2}$	$MC_{eq\ 1}$
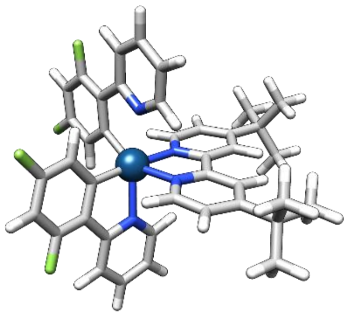 SOC (PBE0) = 3502.76<br>SOC (B3LYP) = 3507.02	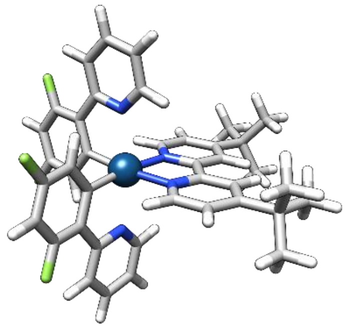 SOC (PBE0) = 3442.92<br>SOC (B3LYP) = 3102.19	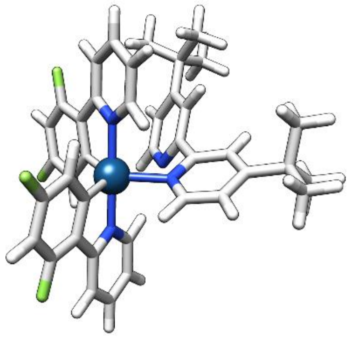 SOC (PBE0) = 1231.65<br>SOC (B3LYP) = 1826.56

[Ir(ppy)$_2$(pyim)]$^{+}$ ref. 18-19			
$MC_{ax\ 1}$	$MC_{ax\ 2}$	$MC_{eq\ 1}$	$MC_{eq\ 2}$
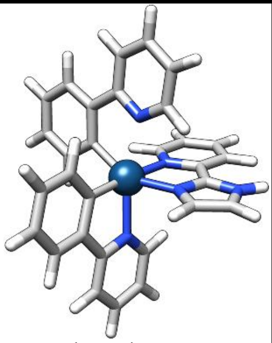 SOC (PBE0) = 1224.23<br>SOC (B3LYP) = 3559.18	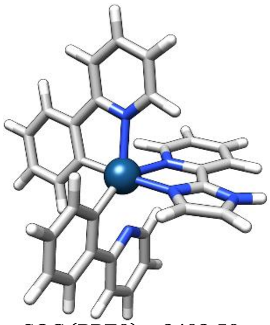 SOC (PBE0) = 3402.50<br>SOC (B3LYP) = 3362.21	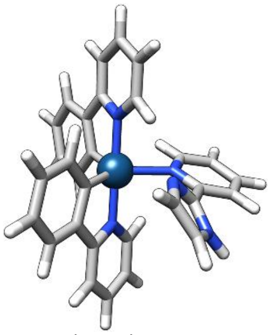 SOC (PBE0) = 1733.03<br>SOC (B3LYP) = 1939.37	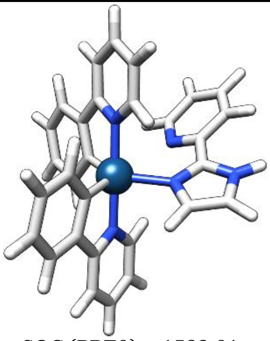 SOC (PBE0) = 1532.01<br>SOC (B3LYP) = 1754.63

[Ir(ppy)$_2$(pyMebim)]$^{+}$ ref. 19			
$MC_{ax\ 1}$	$MC_{ax\ 2}$	$MC_{eq\ 1}$	$MC_{eq\ 2}$
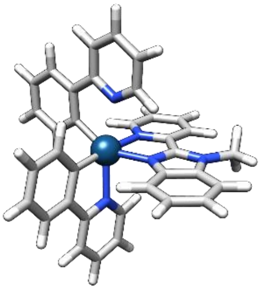 SOC (PBE0) = 3512.28<br>SOC (B3LYP) = 3484.94	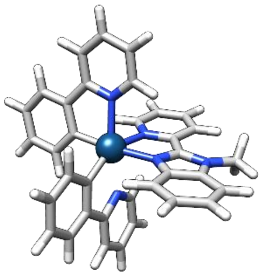 SOC (PBE0) = 3497.86<br>SOC (B3LYP) = 3502.53	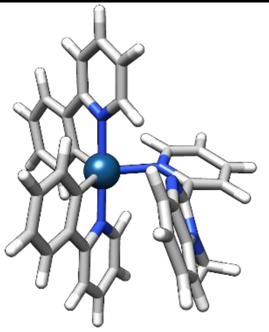 SOC (PBE0) = 1311.46<br>SOC (B3LYP) = 1562.65	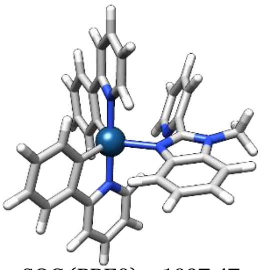 SOC (PBE0) = 1097.47<br>SOC (B3LYP) = 1345.07

[Ir(ppy)$_2$(PIT)]$^{+}$ ref. 20			
$MC_{ax\ 1}$	$MC_{ax\ 2}$	$MC_{eq\ 1}$	$MC_{eq\ 2}$
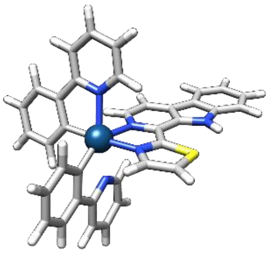 SOC (PBE0) = 3385.49<br>SOC (B3LYP) = 3360.87	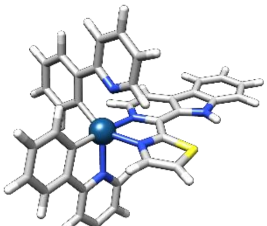 SOC (PBE0) = 3481.33<br>SOC (B3LYP) = 3460.07	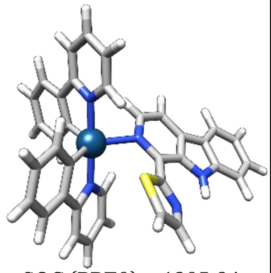 SOC (PBE0) = 1205.84<br>SOC (B3LYP) = 1900.99	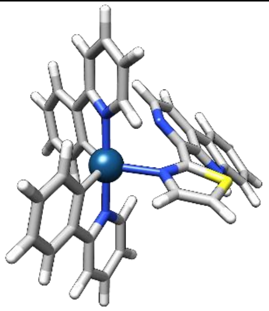 SOC (PBE0) = 1364.10<br>SOC (B3LYP) = 2023.83

$[Ir(ppy)_2(MePIT)]^{+}$ [ref. 20]			
$MC_{ax\,1}$	$MC_{ax\,2}$	$MC_{eq\,1}$	$MC_{eq\,2}$
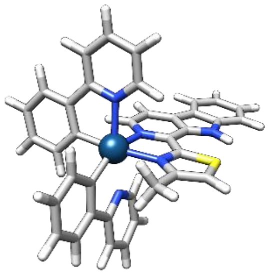<br>SOC (PBE0) = 3484.68<br>SOC (B3LYP) = 3508.76	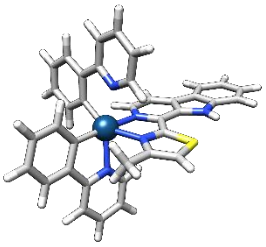<br>SOC (PBE0) = 3453.01<br>SOC (B3LYP) = 3453.19	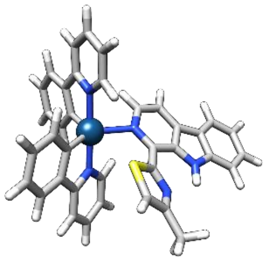<br>SOC (PBE0) = 1626.13<br>SOC (B3LYP) = 1904.12	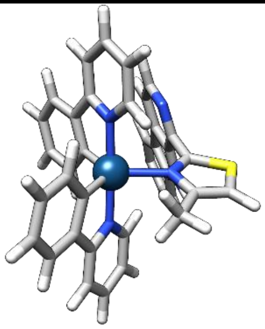<br>SOC (PBE0) = 1226.82<br>SOC (B3LYP) = 1500.36
$[Ir(ppy)_2(PhPIT)]^{+}$ [ref. 20]			
$MC_{ax\,1}$	$MC_{ax\,2}$	$MC_{eq\,1}$	$MC_{eq\,2}$
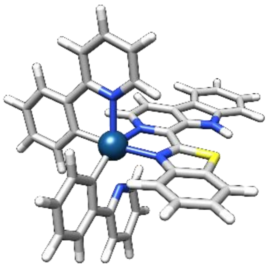<br>SOC (PBE0) = 3478.68<br>SOC (B3LYP) = 3497.71	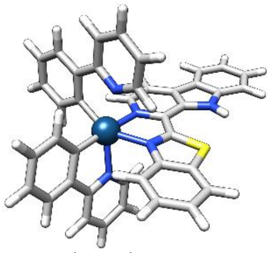<br>SOC (PBE0) = 3426.45<br>SOC (B3LYP) = 3427.13	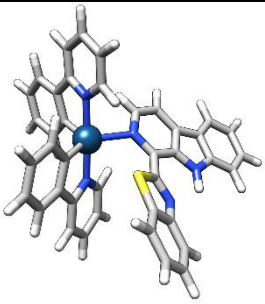<br>SOC (PBE0) = 1643.32<br>SOC (B3LYP) = 1933.49	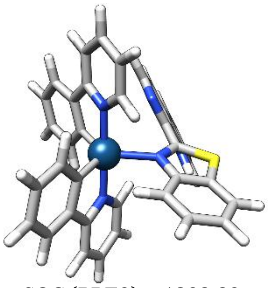<br>SOC (PBE0) = 1293.39<br>SOC (B3LYP) = 1855.36

Figure S14: Optimized geometries calculated for the ^{3}MC minima (axial and equatorial) of a series of Ir(III) complexes. The value of the SOC (in cm^{-1}) between the ^{3}MC and S_0 states computed at the SOC-TDDFT PBE0/(ZORA-def2-TZVP + ZORA-def2-SVP level, SOC(PBE0), and at the SOC-TDDFT B3LYP/(ZORA-def2-TZVP + ZORA-def2-SVP level, SOC(B3LYP), is also reported.

Ru(II) complexes		
$[Ru(bpy)_2(PIT)]^{+2}$ [ref. 19]		
$MC_{ax\ 1}$	$MC_{ax\ 2}$	$MC_{ax\ 3}$
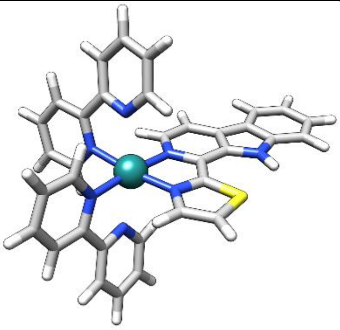 SOC (PBE0) = 1043.75<br>SOC (B3LYP) = 1042.01	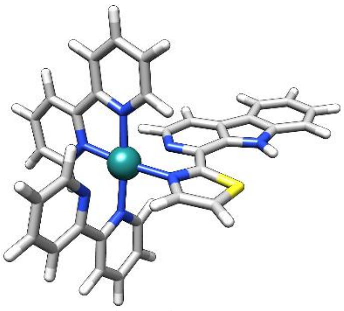 SOC (PBE0) = 1030.54<br>SOC (B3LYP) = 1023.59	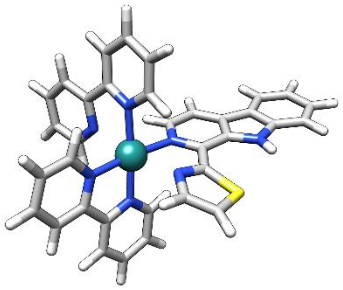 SOC (PBE0) = 1030.08<br>SOC (B3LYP) = 1024.18
$[Ru(bpy)_2(MePIT)]^{+2}$ [ref. 19]		
$MC_{ax\ 1}$	$MC_{ax\ 2}$	$MC_{ax\ 3}$
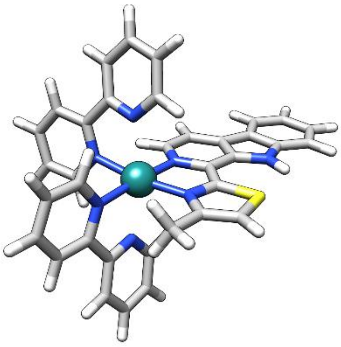 SOC (PBE0) = 1055.20<br>SOC (B3LYP) = 1053.75	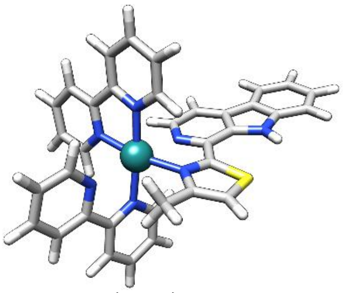 SOC (PBE0) = 1034.64<br>SOC (B3LYP) = 1034.36	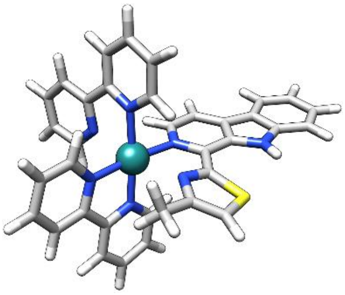 SOC (PBE0) = 1033.23<br>SOC (B3LYP) = 1025.85
$[Ru(bpy)_2(PhPIT)]^{+2}$ [ref. 19]		
$MC_{ax\ 1}$	$MC_{ax\ 2}$	$MC_{ax\ 3}$
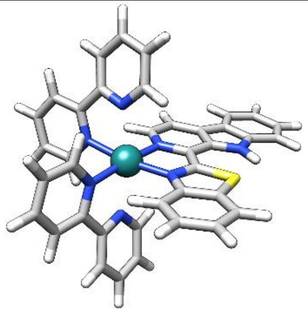 SOC (PBE0) = 1055.29<br>SOC (B3LYP) = 1056.90	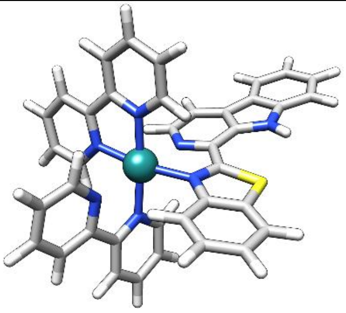 SOC (PBE0) = 1031.08<br>SOC (B3LYP) = 1030.73	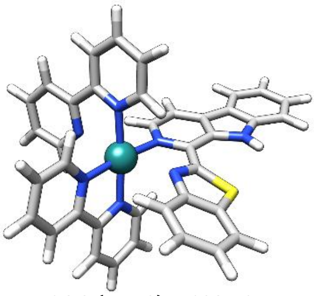 SOC (PBE0) = 1025.97<br>SOC (B3LYP) = 1020.46
$[Ru(TAP)_2(PIT)]^{+2}$ [ref. 19]		
$MC_{ax\ 1}$	$MC_{ax\ 2}$	$MC_{ax\ 3}$
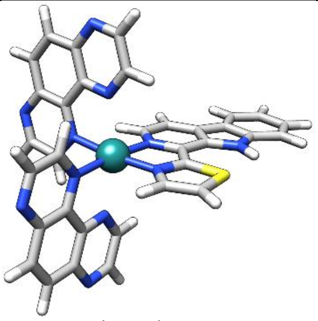 SOC (PBE0) = 1058.45<br>SOC (B3LYP) = 1054.32	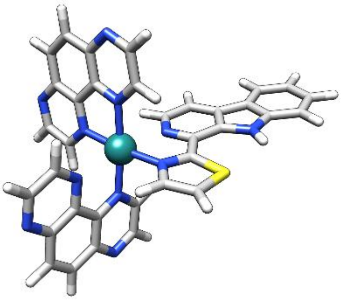 SOC (PBE0) = 1040.52<br>SOC (B3LYP) = 1029.33	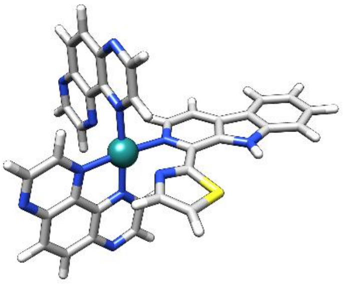 SOC (PBE0) = 1049.81<br>SOC (B3LYP) = 1039.42

[Ru(TAP)$_2$(MePIT)]$^{+2}$ ref. 19		
$MC_{ax\ 1}$	$MC_{ax\ 2}$	$MC_{ax\ 3}$
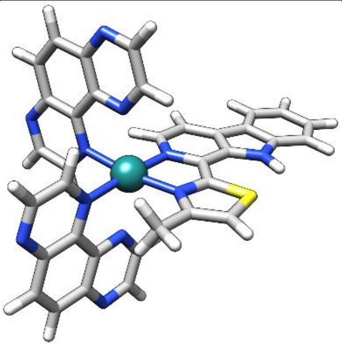 SOC (PBE0) = 1054.28<br>SOC (B3LYP) = 1051.60	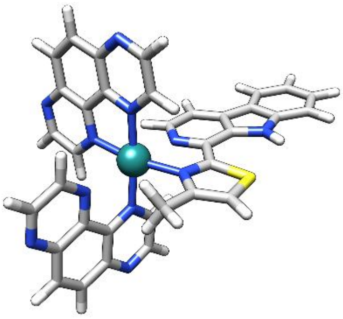 SOC (PBE0) = 1058.09<br>SOC (B3LYP) = 1054.79	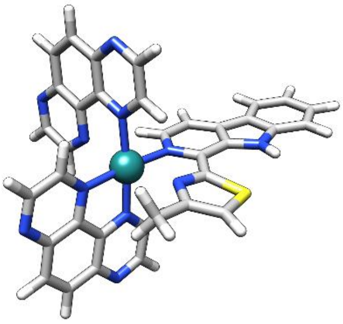 SOC (PBE0) = 1046.21<br>SOC (B3LYP) = 1034.79
[Ru(TAP)$_2$(PhPIT)]$^{+2}$ ref. 19		
$MC_{ax\ 1}$	$MC_{ax\ 2}$	$MC_{ax\ 3}$
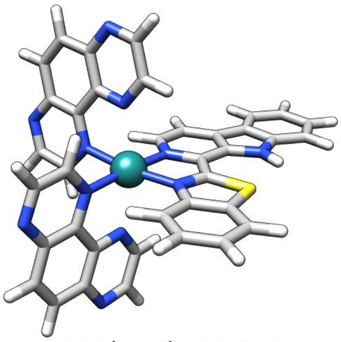 SOC (PBE0) = 1052.69<br>SOC (B3LYP) = 1050.88	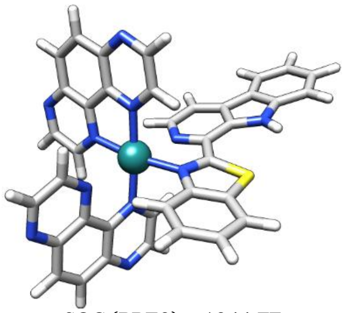 SOC (PBE0) = 1044.77<br>SOC (B3LYP) = 1037.49	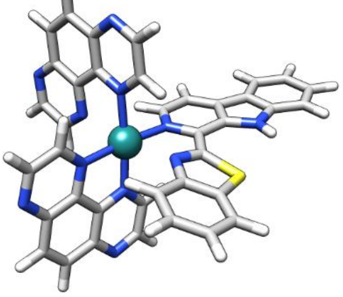 SOC (PBE0) = 1046.74<br>SOC (B3LYP) = 1035.11

Figure S15: Optimized geometries calculated for the ^{3}MC minima of a series of Ru(II) complexes. The value of the SOC (in cm^{-1}) between the ^{3}MC and S_0 states computed at the SOC-TDDFT PBE0/(ZORA-def2-TZVP + ZORA-def2-SVP level, SOC(PBE0), and at the SOC-TDDFT B3LYP/(ZORA-def2-TZVP + ZORA-def2-SVP level, SOC(B3LYP), is also reported.

References

(1) Flamigni, L.; Barbieri, A.; Sabatini, C.; Ventura, B.; Barigelletti, F. Photochemistry and Photophysics of Coordination Compounds: Iridium. *Top. Curr. Chem.* **2007**, *281*, 143–203.

(2) Bhatt, V. Chapter 1 Basic Coordination Chemistry *Essentials of Coordination Chemistry*; Bhatt, V., Ed.; Academic Press, 2016; pp 1−35.

(3) Lasorne, N.; Worth, G.; Robb, M. Excited-state dynamics *Wiley Interdiscip. Rev. Comput. Mol. Sci.* **2011**, *1*, 460-475.

(4) Domcke, W.; Yarkony, D. R.; Köppel, H. Conical Intersections; World Scientific Publishing Co. Pte. Ltd.: Singapore, Japan, **2004**.

(5) Marian, C. M. Understanding and Controlling Intersystem Crossing in Molecules. *Annu. Rev. Phys. Chem.* **2020**, *72*, 617–640.

(6) Neese, F. Software Update: The ORCA Program System—Version 5.0. *Wiley Interdiscip. Rev. Comput. Mol. Sci.***2022**, *12*, e1606.

(7) Adamo, C.; Barone, V. Toward Reliable Density Functional Methods without Adjustable Parameters: The PBE0 Model. *J. Chem. Phys*. **1999**, *110*, 6158−6170.

(8) Van Wüllen, C. Molecular Density Functional Calculations in the Regular Relativistic Approximation: Method, Application to Coinage Metal Diatomics, Hydrides, Fluorides and Chlorides, and Comparison with First-Order Relativistic Calculations. *J. Chem. Phys.* **1998**, *109*, 392–399.

(9) Heß, B. A.; Marian, C. M.; Wahlgren, U.; Gropen, O. A Mean-Field Spin-Orbit Method Applicable to Correlated Wavefunctions. *Chem. Phys. Lett.* **1996**, *251*, 365–371.

(10) Weigend, F.; Ahlrichs, R. Balanced Basis Sets of Split Valence, Triple Zeta Valence and Quadruple Zeta Valence Quality for H to Rn: Design and Assessment of Accuracy. *Phys. Chem. Chem. Phys.* **2005**, *7*, 3297.

(11) Pantazis, D. A.; Chen, X.-Y.; Landis, C. R.; Neese, F. All-Electron Scalar Relativistic Basis Sets for Third-Row Transition Metal Atoms. *J. Chem. Theory Comput.* **2008**, *4*, 908–919.

(12) Dergachev, V. D.; Rooein, M.; Dergachev, I. D.; Lykhin, A. O.; Mauban, R. C.; Varganov, S. A. NAST: Nonadiabatic Statistical Theory Package for Predicting Kinetics of Spin-Dependent Processes. *Top. Curr. Chem.* **2022**, *380*, 1–25.

(13) Fdez. Galván, I.; Vacher, M.; Alavi, A.; Angeli, C.; Aquilante, F.; Autschbach, J.; Bao, J. J.; Bokarev, S. I.; Bogdanov, N. A.; Carlson, R. K.; Chibotaru, L. F.; Creutzberg, J.; Dattani, N.; Delcey, M. G.; Dong, S. S.; Dreuw, A.; Freitag, L.; Frutos, L. M.; Gagliardi, L.; Gendron, F.; Giussani, A.; González, L.; Grell, G.; Guo, M.; Hoyer, C. E.; Johansson, M.; Keller, S.; Knecht, S.; Kovačević, G.; Källman, E.; Li Manni, G.; Lundberg, M.; Ma, Y.; Mai, S.; Malhado, J. P.; Malmqvist, P. Å.; Marquetand, P.; Mewes, S. A.; Norell, J.; Olivucci, M.; Oppel, M.; Phung, Q. M.; Pierloot, K.; Plasser, F.; Reiher, M.; Sand, A. M.; Schapiro, I.; Sharma, P.; Stein, C. J.; Sørensen, L. K.; Truhlar, D. G.; Ugandi, M.; Ungur, L.; Valentini, A.; Vancoillie, S.; Veryazov, V.; Weser, O.; Wesołowski, T. A.; Widmark, P. O.; Wouters, S.; Zech, A.; Zobel, J. P.; Lindh, R. OpenMolcas: From Source Code to Insight. *J Chem. Theory Comput.* **2019**, *15*, 5925–5964.

(14) Bokarev, S. I.; Bokareva, O. S.; Kühn, O. Electronic Excitation Spectrum of the Photosensitizer $[Ir(Ppy)_2(Bpy)]^+$. *J. Chem. Phys.* **2012**, *136*, 214305.

(15) Robb, M. A.; Garavelli, M.; Olivucci, M.; Bernardi, F. In Reviews in Computational Chemistry; Lipkowitz K. B. and Boyd, D. B. Eds., Wiley-VCH, New York, **2000**, Vol 15, pp 87-212.

(16) Soriano-Díaz, I.; Ortí, E.; Giussani, A. On the Importance of Equatorial Metal-Centered Excited States in the Photophysics of Cyclometallated Ir(III) Complexes. *Dalton Trans.* **2023**, *52*, 10437–10447.

(17) Costa, R. D.; Monti, F.; Accorsi, G.; Barbieri, A.; Bolink, H. J.; Ortí, E.; Armaroli, N. Pho-to-physical Properties of Charged Cyclometalated Ir(III) Complexes: A Joint Theoretical and Experimental Study. *Inorg. Chem.* **2011**, *50*, 7229–7238.

(18) Soriano-Díaz, I.; Ortí, E.; Giussani, A. Predicting Nonradiative Decay Rate Constants of Cyclometalated Ir(III) Complexes. *Inorg. Chem.* **2024**, *63*, 16600–16604.

(19) Martínez-Alonso, M.; Cerdá, J.; Momblona, C.; Pertegás, A.; Junquera-Hernández, J. M.; Heras, A.; Rodríguez, A. M.; Espino, G.; Bolink, H.; Ortí, E. Highly Stable and Efficient Light-Emitting Electrochemical Cells Based on Cationic Iridium Complexes Bearing Aryla-zole Ancillary Ligands. *Inorg. Chem.* **2017**, *56*, 10298–10310.

(20) Sanz-Villafruela, J.; Bermejo-Casadesus, C.; Zafon, E.; Martínez-Alonso, M.; Durá, G.; Heras, A.; Soriano-Díaz, I.; Giussani, A.; Ortí, E.; Tebar, F.; Espino, G.; Massaguer, A. In-sights into the Anticancer Photodynamic Activity of Ir(III) and Ru(II) Polypyridyl Com-plexes Bearing β-Carboline Ligands. *Eur. J. Med. Chem.* **2024**, *276*, 116618.
