# Supplementary material for: On the Role of Spin–Orbit Coupling and State-Crossing Topography in the Nonradiative Decay of Ir(III) Complexes
Source: J Phys Chem Lett. 2025 Aug 25;16(35):9004–10. doi: 10.1021/acs.jpclett.5c01776 (PMC12415876; doi:10.1021/acs.jpclett.5c01776)
Supplement: Supplementary file 2 [file jz5c01776_si_002.pdf]

jz-2025-01776a.R1

Name: Peer Review Information for "The role of spin-orbit coupling and state-crossing topography in the non-radiative decay of Ir(III) complexes"

First Round of Reviewer Comments

Reviewer: 1

Comments to the Author

In this study, the authors revisit the nonradiative decay pathways of the [Ir(ppy)2(bpy)]+ complex and challenge the prevailing assumption that triplet metal-centered (triplet MC) states efficiently mediate nonradiative decay to the ground state via strong spin-orbit coupling (SOC) and a minimum-energy crossing point (MECP). This mechanistic framework is re-examined through a combination of theoretical analyses, including a detailed investigation of the topography of the triplet MC/S0 MECP, the interplay between SOC and nonadiabatic couplings (NACs) in both spin-pure and spin-mixed representations, and kinetic modeling based on nonadiabatic transition state theory (NA-TST). The authors conclude that in order to suppress nonradiative deactivation, the triplet MC state must be destabilized so that it no longer possesses a well-defined local minimum on the excited-state potential energy surface. This theoretical insight highlights the importance of rigorously characterizing the excited-state reaction pathway when designing phosphorescent metal complexes, making the study meaningful and relevant. However, several issues need to be addressed before the manuscript can be considered for publication. Accordingly, I recommend major revisions. The following points summarize the main concerns:

1. While the authors propose a mechanism involving the triplet MC state, they do not discuss how to avoid this decay pathway in practice. The conclusion that destabilizing the triplet MC state is beneficial remains abstract and offers little guidance for molecular design. Concrete suggestions for avoiding population of the triplet MC minimum should be provided.

2. Although the manuscript includes schematic diagrams of the potential energy surface connecting the T1 and triplet MC states, it does not provide computed barrier heights or transition-state structures. Without such data, it is unclear whether the population transfer from T1 to triplet MC is kinetically feasible. At the very least, estimated activation barriers or plausible TS geometries should be included.

3. Excitation energies and equilibrium structures of T1 and triplet MC states are not presented in the main text or the Supporting Information (SI). All structural and energetic data used in the discussion should be compiled and made available in the SI.

4. Molecular orbitals associated with key electronic transitions (e.g., MLCT) are not shown. These are necessary not only to understand the nature of the excited states but also to analyze the electron motion during the excited-state reaction. These MO visualizations should be included in the SI.

5. The study employs global hybrid functionals (B3LYP, PBE0), which are known to inadequately describe charge-transfer excitations and orbital energies. The rationale for choosing such functionals should be explained. Prior studies have pointed out the limitations of global hybrids in accurately modeling MLCT states in metal complexes [e.g., Niehaus et al., RSC Adv., 5, 63318 (2015)].

Reviewer: 2

Comments to the Author

Giussani and co-workers discuss the role of spin-orbit coupling (SOC) in the non-radiative decay of Ir(III) complexes. They show how the mixing of pure spin states of different spin under the effect of SOC effects the decay mechanisms. Namely, they demonstrate that the interpretation of the decay process is qualitatively different when SOC is taken into account in the electronic eigenstates and not just in the operator matrix elements that induce transitions. The results are highly important for our understanding of non-radiative decay and demonstrate that in the case of heavy elements such as Ir, the discussion of

electronic structure in terms of pure spin states is not correct due to the strong SOC effects. The paper is clearly written (although the ESI could benefit from additional proofreading as there are some unclear sentence structures).

While I agree with the authors' conclusions and wish to see this work published, there is a major issue related to the lack of experimental or quantum-chemical evidence for the conclusions. This issue should be resolved before the manuscript can be published in the Journal of Physical Chemistry Letters.

The main issue with the manuscript is that the conclusions rely entirely on the theoretical derivations presented in the manuscript. As the authors note in the text, experimental evidence for the proposed results is extremely difficult to obtain. Therefore, the conclusions should be supported by ab initio calculations that fully reproduce the features of the potential energy surface that are necessary for supporting the conclusions. The authors state that they are currently considering the possibility of carrying out multireference calculations. Although the systems are relatively large, and the strong metal–ligand covalency and charge-transfer nature of the electronic states make such calculations demanding and time-consuming, they are still feasible. A calculation that includes the 5d orbitals, a double-shell of d orbitals and the Eg-symmetric ligand sigma orbitals (i.e., CASSCF(10,12)) is still possible, and an additional perturbative dynamic electron correlation treatment at CASPT2, NEVPT2, etc. level is also possible. Treament of SOC using the QDPT in Orca or SOC-RASSI in (Open)Molcas should then provide at least a qualitatively correct features of the potential energy surface that the authors need to support their conclusions. Without such calculations the paper should be taken as a more speculative paper, that is definitely worth publishing, but not in J. Chem. Phys. Lett.

Therefore, I suggest that the authors carry out multireference calculations to provide ab initio support for their conclusions, or alternatively resubmit the manuscript to a journal such as J. Phys. Chem. A or Int. J. Quantum. Chem.

Reviewer: 3

Comments to the Author

he manuscript is devoted to a theorical investigation about the electronic deactivation of Ir(III) complexes. In particular, the author discuss about the possibility of observing ultrafast decay to the ground state S0 from a local minimum of the first excited state T1, involving a cleavage of one of the ML bonds. The discussion made by the authors is interesting and physically sound. Nevertheless, the manuscript presents major problems that prevent publication in the current form. Here are my remarks.

1) No attempt is presented of a computational evaluation of the intersystem crossing rate for the system considered in the manuscript. I understand that the evaluation of the relevant electronic quantities is computationally challenging, but some attempt is needed.

2) Page 3 of the manuscript, as well as section S3 of the SI, are devoted to show how the equation of motion for nuclear wavepackets is derived from the TDSE. Now, this is textbook matter. Moreover, it is not relevant for the manuscript. Finally, it is wrongs. In particular, the expression for the Born-Oppenheimer coupling (operator "lambda" in equation 2) is not correct: even admetting to neglect the second derivative coupling terms, one has still to multiply the expression on the RHS by the the nuclear linear momentum operator divided by the nuclear mass.

3) According to authors, if the three components of the T1 triplet are explicitly considered, then the S0/T1 splitting at the MECP should be divided by two. I agree that the tree component of the triplet can be linearly combined such that only one interacts with S0. But then, one can have a direct S-T population transfer only through the component which interacts with the singlet, not from the other two.

Therefore, the S0/T1 splitting does not change, even taking into account the three components.

4) If I understand correctly, the NA-TST approach the authors would intend to use is based on the Landau-Zener equation (eq. S21). However, special care must be taken in this respect, when considering a sloped avoided crossing. In fact, two necessary conditions for eq. S21 to be valid are (i) the nuclear velocity is approximately constant during the crossing of the strong interaction region, and (ii) the region of strong interaction is fully traversed. Both (i) and (ii) are not fulfilled if the systems arrives at the sloped avoided crossing with a low velocity (i.e., if the denominator under the square root of eq. S21 is close to zero).

Author's Response to Peer Review Comments:

Editor: *The Journal Physical Chemistry Letters.*

Valencia, July 30th 2025

Dear Editor,

Attached please find the revised version of our paper entitled: "***The role of spin-orbit coupling and state-crossing topography in the non-radiative decay of Ir(III) complexes***", by Iván SorianoDíaz, Ilya D. Dergachev, Sergey A. Varganov, Enrique Ortí, and Angelo Giussani. We thank you and the reviewers for providing valuable comments. In separate pages, we list our replies to the reviewers' suggestions and the changes introduced in the manuscript. We hope that the current version is suitable for publication in *The Journal Physical Chemistry Letters.*

With our best regards, Dr.
Angelo Giussani.

Reviewer 1.

1. "*While the authors propose a mechanism involving the triplet MC state, they do not discuss how to avoid this decay pathway in practice. The conclusion that destabilizing the triplet MC state is beneficial remains abstract and offers little guidance for molecular design. Concrete suggestions for avoiding population of the triplet MC minimum should be provided*."

Authors reply. The reviewer is asking a very important question, yet very difficult to address. Based on our hypothesis, according to which the involvement of ^{3}MC state in the non-radiative decay of an Ir(III) complex will be dictated not only by the ability of populating the corresponding ^{3}MC minimum but also by the ability to remain trapped in such a minimum, we can propose the following suggestion (appearing in page 8 of the revised manuscript) in order to minimize the ^{3}MC mediated non-radiative decay:

We can then suggest that in order to minimize the non-radiative decay mediated by ^{3}MC states, a key design strategy will be to avoid complexes that in their ^{3}MC minima display stabilizing interactions not present in the emitting structure. Such interactions could in fact determine a sizable barrier from the ^{3}MC to the T_1 minimum and in turn the trapping of the population in the ^{3}MC state.

2. *"Although the manuscript includes schematic diagrams of the potential energy surface connecting the T1 and triplet MC states, it does not provide computed barrier heights or transition-state structures. Without such data, it is unclear whether the population transfer from T1 to triplet MC is kinetically feasible. At the very least, estimated activation barriers or plausible TS geometries should be included."*

3. *"Excitation energies and equilibrium structures of T1 and triplet MC states are not presented in the main text or the Supporting Information (SI). All structural and energetic data used in the discussion should be compiled and made available in the SI."*

4. *"Molecular orbitals associated with key electronic transitions (e.g., MLCT) are not shown. These are necessary not only to understand the nature of the excited states but also to analyze the electron motion during the excited-state reaction. These MO visualizations should be included in the SI."*

Authors reply to questions 2,3,4. The next sentence is now included in page 2 of the revised manuscript:

The energies, structures and orbital analysis of the described paths involving the $^3MC_{ax}$ and $^3MC_{eq}$ states are present in Section S3. These data have been previously obtained at the B3LYP/def2-SVP CPCM (CH_2Cl_2) level,[13] and now also using the PBE0 functional.

The new Section 3 now reports all the requested data, which allow to analyze the energy involved in the process, the geometrical deformations experimented by the complex, and the nature of the involved electronic states. It is however important to remark that, although such data are fundamental in order to judge the feasibility of the process, the studied ^{3}MC-mediated non-radiative decay paths for the reference system $[Ir(C^\wedge N)_2(N^\wedge N)]^+$ merely constitute an example. Such an example clearly shows the effect and the importance of explicitly considering both the SOC and the topography of the ^{3}MC/S_0 MECP for Ir(III) transition-metal complexes, independently of the relevance of the process for the specific $[Ir(C^\wedge N)_2(N^\wedge N)]^+$ system.

5. *"The study employs global hybrid functionals (B3LYP, PBE0), which are known to inadequately describe charge-transfer excitations and orbital energies. The rationale for choosing such functionals should be explained. Prior studies have pointed out the limitations of global hybrids in accurately modeling MLCT states in metal complexes [e.g., Niehaus et al., RSC Adv., 5, 63318 (2015)]."*

Authors reply. Indeed, as mentioned in the cited article by Niehaus et al, TDDFT in general and global hybrid functionals in particular can be problematic for the description of charge-transfer states, usually leading to an underestimation of their energy. All our energies are however obtained using DFT, and not TDDFT, since we are working with the S_0 and T_1 states. The only TDDFT calculations were the ones in order to obtain the SOC values, with the main goal of obtaining a qualitative result showing a value of the order of thousands cm^{-1}. We are however now presenting SOC values obtained out from ab-initio RASSCF-RASSI calculations, as now specified in page 6 of the revised manuscript:

In the present work, a first RASSCF calculation was performed on the PBE0-optimized $^3MC_{ax}/S_0$ MECP, taking as a reference the work of Bokarev et al.,[43] followed by the computation of the corresponding spin-mixed states using the RASSI code of OpenMolcas (see Section S7).[44,45] From such a calculation we obtained a SOC between the $^3MC_{ax}$ and S_0 states of 3028 cm^{-1} (0.37 eV), in agreement with the TDDFT value of 3400 cm^{-1} (0.42 eV), and a splitting of the spin-mixed states of 0.76 eV, indeed equal to roughly two times the SOC value.

Regarding the choice of functionals, this is always a delicate question. Our choice is dictated by our experience with B3LYP and our recent work comparing B3LYP with PBE0 (Inorg. Chem. 2024, 63, 16600−16604) showing how PBE0 provide higher energies for the ^{3}MC states. It is however important to remark that while the choice of the specific functional can be critical for obtaining the accurate energies and in turn the rate constants, the fundamental fact on which our conclusions are based will not change, which is the presence of sloped ^{3}MC/S_0 MECPs characterized by a SOC value of the order of thousands cm^{-1}.

Reviewer 2

"The main issue with the manuscript is that the conclusions rely entirely on the theoretical derivations presented in the manuscript. As the authors note in the text, experimental evidence for the proposed results is extremely difficult to obtain. […] A calculation that includes the 5d

orbitals, a double-shell of d orbitals and the Eg-symmetric ligand sigma orbitals (i.e., CASSCF(10,12)) is still possible, and an additional perturbative dynamic electron correlation treatment at CASPT2, NEVPT2, etc. level is also possible. Treament of SOC using the QDPT in Orca or SOC-RASSI in (Open)Molcas should then provide at least a qualitatively correct features of the potential energy surface that the authors need to support their conclusions. Therefore, the conclusions should be supported by ab initio calculations that fully reproduce the features of the potential energy surface that are necessary for supporting the conclusions."

Authors reply. The two features of the potential energy surface that support our conclusion are: the existence of sloped MECPs between the ^{3}MC and S_0 states, and that the SOC between the ^{3}MC and S_0 states is significantly large. As explain in Section S2, the presence of sloped ^{3}MC/S_0 MECPs is a direct consequence of the electronic structure of this type of complexes. Independently of the employed electronic structure approach, ^{3}MC states exist and tend to dissociate a coordination bond. This in turn determines a massive rise of the ground-state energy, eventually leading to the interchange of the energy order of the S_0 and ^{3}MC, which implies a crossing. The fact that such a crossing will describe a broken bond, which is instead present both in the T_1 and S_0 minima, determines the sloped topography of the MECP. In order to prove the second feature, following the reviewer suggestion, we performed RASSCF calculations and subsequent RASSI-SOC computations with OpenMolcas. The employed active space is slightly different from that suggested by the reviewer. While we managed to include 5d orbitals, the Eg-symmetric ligand sigma orbitals and main orbitals of the ligands in the active space, given the limited time, we could not include a second double-shell of d orbitals (see Section S7) and we could not obtain the RASPT2 energies. A double-shell of d orbitals should in principle be more relevant for first row transition-metal complexes, although we are aware that in the work of Bokarev et al. (J. Chem. Phys. 136, 214305 (2012)) their importance was indeed recognized for the correct description of MLCT states, while their contribution for MC states was not described. The performed calculation, however, confirms the very high SOC value characterizing the ^{3}MC and S_0 states and the fact that the inclusion of the SOC leads to the energy gap between the two adiabatic states twice the SOC value, which justifies the linear crossing model employed to calculate the Landau-Zener transition probability. We do not expect that the inclusion of the second shell of d orbitals and the RASPT2 correction will lead to qualitative changes. The new RASSCF results are discussed revised manuscript and in Section S7 of the SI. The following explanatory paragraph has been included in the new version of the manuscript at page 6.

In the present work, a first RASSCF calculation was performed on the PBE0-optimized $^3MC_{ax}/S_0$ MECP, taking as a reference the work of Bokarev et al.,[43] followed by the computation of the corresponding spin-mixed states using the RASSI code of OpenMolcas (see Section S7).[44,45] From such a calculation we obtained a SOC between the $^3MC_{ax}$ and S_0 states of 3028 cm^{-1} (0.37 eV), in agreement with the TDDFT value of 3400 cm^{-1} (0.42 eV), and a splitting of the spin-mixed states of 0.76 eV, indeed equal to roughly two times the SOC value.

Reviewer 3.

1. *"No attempt is presented of a computational evaluation of the intersystem crossing rate for the system considered in the manuscript. I understand that the evaluation of the relevant electronic quantities is computationally challenging, but some attempt is needed."*

Authors reply. We have now evaluated the non-radiative decay constants associated with the non-radiative decay mediated by the ^{3}MC states using non-adiabatic transition state theory (NAST) and the Landau-Zener and Zhu-Nakamura transition probabilities. The corresponding discussion is now present in page 5 of the new manuscript and in Section S6:

[…]This conclusion is confirmed by computing the corresponding probability and non-radiative decay rate constant, $k_{nr}(T)$, at the $^3MC_{ax}/S_0$ MECP and $^3MC_{eq}/S_0$ MECP of $[Ir(ppy)_2(bpy)]^+$ using the non-adiabatic statistical theory (NAST, see Section S6).[28,42] Employing the Landau-Zener equation (S22-S23), the $k_{nr}(T)$ values for the non-radiative decay path associated with the $^3MC_{ax}/S_0$ and $^3MC_{eq}/S_0$ MECPs are 1.81×10^{-7} and 2.32×10^{-2} s^{-1}, respectively. Both values are significantly lower than those predicted by transition state theory (5.37 and 5.50×10^2 s^{-1}, respectively) and lower than the experimental total non-radiative rate constant (around 10^6 s^1). Moreover, the $k_{nr}(T)$ associated with the $^3MC_{ax}/S_0$ is five orders of magnitude lower than the rate constant for the $^3MC_{eq}/S_0$ MECP. We hypothesize that this difference can be related to the slightly higher energy barrier to reach the $^3MC_{ax}/S_0$ MECP than the $^3MC_{eq}/S_0$ MECP (Figure S3), but also to the larger SOC at the $^3MC_{ax}/S_0$ MECP compared to $^3MC_{eq}/S_0$ MECP (3400 vs 1676 cm^1). In fact, for the characterized sloped MECPs, a stronger SOC results in a larger energy gap between the spin-adiabatic PESs, leading to a less efficient non-radiative decay. To account for quantum tunneling and the non-linear behavior of reaction path, neglected in the Landau-Zener treatment, we computed the $k_{nr}(T)$ using the Zhu-Nakamura (ZN) transition probability equation for the non-radiative decay path mediated by the $^3MC_{ax}/S_0$ MECP. The rate calculated with ZN (2.02×10^4 s^{-1}) indicates a massive effect of quantum tunneling on overall non-radiative decay rate from the T_1 state, due to low MECP barrier and small reduced mass along the reaction coordinate (see Section S6). It is worth noticing that the ZN rate constant, in principle more accurate that the LZ result, do not only describe the probability of non-radiative decay through MECP (since it includes tunneling). Consequently, the ZN value cannot be taken as direct measure of the non-radiative decay rate through MECP, which is the main subject of the present work.

2. *"Page 3 of the manuscript, as well as section S3 of the SI, are devoted to show how the equation of motion for nuclear wavepackets is derived from the TDSE. Now, this is textbook matter. Moreover, it is not relevant for the manuscript. Finally, it is wrongs. In particular, the expression for the Born-Oppenheimer coupling (operator "lambda" in equation 2) is not correct: even admetting to neglect the second derivative coupling terms, one has still to multiply the expression on the RHS by the nuclear linear momentum operator divided by the nuclear mass."*

Authors reply. We thank the reviewer for this comment. Indeed equation 2 was incorrect. Even if our intention was indeed to approximate the Born-Oppenheimer coupling operator neglecting the second derivative coupling terms, and for that reason we didn't employ the equal symbol in

the original equation 2, still the nuclear linear momentum operator divided by the nuclear mass was missing. We have corrected the issue in what is now equation 2 (and S9 of the SI) and we now present the result of the generalized Hellman-Feynman theorem as a new equation (eq. 3).

Although the presented derivation can be framed as textbook material, we consider it fundamental in order to properly understand the work, so we believe it is convenient to maintain it both in the main text and the SI.

3. “*According to authors, if the three components of the T1 triplet are explicitly considered, then the S0/T1 splitting at the MECP should be divided by two. I agree that the tree component of the triplet can be linearly combined such that only one interacts with S0. But then, one can have a direct S-T population transfer only through the component which interacts with the singlet, not from the other two. Therefore, the S0/T1 splitting does not change, even taking into account the three components*.”

Authors reply. We thank the reviewer for this comment. The original discussion *“… They showed that two of the three sublevels have the same energy as the original T_1 state, whereas the third one increases its energy by a value equal to the SOC, and the spin-mixed S_0 state decreases its energy by a value equal to the SOC (see equations 7 of reference 31). We then conclude that when considering the three sublevels of T_1, the splitting at MECP between the spin-mixed state associated with S_0 and the lowest-energy components of the spin-mixed states associated with T_1, is equal to the SOC value. In our specific example, i.e. the $[Ir(ppy)_2(bpy)]^+$ complex, that corresponds to a 0.42 and 0.21 eV energy splitting at the $^3MC_{ax}/S_0$ and $^3MC_{eq}/S_0$ MECPs, respectively. These energies, although half the value obtained when ignoring the triplet zerofield splitting, are still very high, leading to small NAC (see eq 2)*.” has now been simplified as follow:

They showed that two of the three sublevels have the same energy as the original T_1 state, whereas the third one increases its energy by a value equal to the SOC, and the spin-mixed S_0 state decreases its energy by a value equal to the SOC (see equations 7 of reference 31). Only the last two spin-mixed states are interacting, which again are energetically separated by a value two times their original SOC.

4. “*If I understand correctly, the NA-TST approach the authors would intend to use is based on the Landau-Zener equation (eq. S21). However, special care must be taken in this respect, when considering a sloped avoided crossing. In fact, two necessary conditions for eq. S21 to be valid are (i) the nuclear velocity is approximately constant during the crossing of the strong interaction region, and (ii) the region of strong interaction is fully traversed. Both (i) and (ii) are not fulfilled if the systems arrives at the sloped avoided crossing with a low velocity (i.e., if the denominator under the square root of eq. S21 is close to zero).*”

Authors reply.

The reviewer is correct that in a special case, when the internal energy along the reaction coordinate is smaller or slightly above of the MECP barrier (low velocity along the reaction

coordinate), the Landau-Zener (LZ) transition probability can be inaccurate. To account for this limitation, in addition to calculating the NAST rates using the LZ probability, we also performed calculations with the more sophisticated Zhu-Nakamura (ZN) equations for the sloped crossing. The ZN transition probability accounts for a nonlinear behavior of the reaction path and quantum tunneling through the MECP barrier, predicting a non-zero transition probability even if the strong interaction region is not fully traversed. The following sentences were added to the main text in page 5-6:

To account for quantum tunneling and the non-linear behavior of reaction path, neglected in the Landau-Zener treatment, we computed the $k_{nr}(T)$ using the Zhu-Nakamura (ZN) transition probability equation for the non-radiative decay path mediated by the $^3MC_{ax}/S_0$ MECP. The rate calculated with ZN (2.02×10^4 s^{-1}) indicates a massive effect of quantum tunneling on overall non-radiative decay rate from the T_1 state, due to low MECP barrier and small reduced mass along the reaction coordinate (see Section S6). It is worth noticing that the ZN rate constant, in principle more accurate that the LZ result, do not only describe the probability of non-radiative decay through MECP (since it includes tunneling). Consequently, the ZN value cannot be taken as direct measure of the non-radiative decay rate through MECP, which is the main subject of the present work.

The details of the NAST rate constant calculations with the ZN transition probability were added to Supporting Information in Section S6.

jz-2025-01776a.R2

Name: Peer Review Information for "The role of spin-orbit coupling and state-crossing topography in the non-radiative decay of Ir(III) complexes"

Second Round of Reviewer Comments

Reviewer: 1

Comments to the Author

After reading the authors’ response, I have confirmed that the revisions have been made to an acceptable extent. Therefore, I conclude that the manuscript can be published.

However, I would like to raise a caution regarding the choice of functionals in this study. Although the study involves MLCT calculations, the authors used global hybrid functionals, which are incapable of properly describing charge-transfer excitations. It was acceptable in this case only because the property under investigation, SOC, happens to be relatively insensitive to the choice of functional. Had the study focused on other properties, I would likely have requested a recalculation. For future calculations on systems where charge transfer occurs, such as large-scale systems, I strongly recommend the use of a range-separated hybrid functional.

Reviewer: 3

Comments to the Author

The authors have addressed my comments appropriately, providing quite extensive modifications and additions to the manuscript. I recommend the manuscript for publication

Reviewer: 2

Comments to the Author

The reviewers have satisfactorily addressed the issues I raised.

Author's Response to Peer Review Comments:

Editor: *The Journal Physical Chemistry Letters.*

Valencia, August 15th 2025

Dear Editor,

Attached please find the revised version of our paper entitled: "***The role of spin-orbit coupling and state-crossing topography in the non-radiative decay of Ir(III) complexes***", by Iván SorianoDíaz, Ilya D. Dergachev, Sergey A. Varganov, Enrique Ortí, and Angelo Giussani.

All mentioned non-scientific changes have been made. We hope that the current version is suitable for publication in *The Journal Physical Chemistry Letters.*

With our best regards, Dr.
Angelo Giussani.
